# The Bicolored White-Toothed Shrew *Crocidura leucodon* (HERMANN 1780) Is an Indigenous Host of Mammalian Borna Disease Virus

**DOI:** 10.1371/journal.pone.0093659

**Published:** 2014-04-03

**Authors:** Ralf Dürrwald, Jolanta Kolodziejek, Herbert Weissenböck, Norbert Nowotny

**Affiliations:** 1 IDT Biologika GmbH (IDT), Dessau-Roβlau, Germany; 2 Viral Zoonoses, Emerging and Vector-Borne Infections Group, Institute of Virology, Department of Pathobiology, University of Veterinary Medicine Vienna, Vienna, Austria; 3 Institute of Pathology and Forensic Veterinary Medicine, Department of Pathobiology, University of Veterinary Medicine Vienna, Vienna, Austria; 4 Department of Microbiology and Immunology, College of Medicine and Health Sciences, Sultan Qaboos University, Muscat, Oman; National Institutes of Health. National Institute of Allergy and Infectious Diseases, Division of Clinical Research, United States of America

## Abstract

Borna disease (BD) is a sporadic neurologic disease of horses and sheep caused by mammalian Borna disease virus (BDV). Its unique epidemiological features include: limited occurrence in certain endemic regions of central Europe, yearly varying disease peaks, and a seasonal pattern with higher disease frequencies in spring and a disease nadir in autumn. It is most probably not directly transmitted between horses and sheep. All these features led to the assumption that an indigenous virus reservoir of BDV other than horses and sheep may exist. The search for such a reservoir had been unsuccessful until a few years ago five BDV-infected shrews were found in a BD-endemic area in Switzerland. So far, these data lacked further confirmation. We therefore initiated a study in shrews in endemic areas of Germany. Within five years 107 shrews of five different species were collected. BDV infections were identified in 14 individuals of the species bicolored white-toothed shrew (*Crocidura leucodon*, HERMANN 1780), all originating from BD-endemic territories. Immunohistological analysis showed widespread distribution of BDV antigen both in the nervous system and in epithelial and mesenchymal tissues without pathological alterations. Large amounts of virus, demonstrated by presence of viral antigen in epithelial cells of the oral cavity and in keratinocytes of the skin, may be a source of infection for natural and spill-over hosts. Genetic analyses reflected a close relationship of the BDV sequences obtained from the shrews with the regional BDV cluster. At one location a high percentage of BDV-positive shrews was identified in four consecutive years, which points towards a self-sustaining infection cycle in bicolored white-toothed shrews. Analyses of behavioral and population features of this shrew species revealed that the bicolored white-toothed shrew may indeed play an important role as an indigenous host of BDV.

## Introduction

Borna disease viruses (BDVs) are nonsegmented negative stranded RNA viruses of the family *Bornaviridae* within the order *Mononegavirales* which replicate and transcribe in the nucleus of infected cells and use cellular splicing for generating some mRNAs [1]–[3]. The viruses are noncytolytic, have a high tropism for cells of the central nervous system and infect mammals [4], birds [5]–[8], and have even been found in a snake venome gland which could be a sign of natural infection of reptiles [9]. A large number of species harbors BDV-like elements in their genomes indicating these viruses emerged long ago [9]–[12]. Clinical symptoms are mainly caused by immunopathological reactions of antiviral CD8 T cells to infected cells [13], [14]. Two disease complexes are known which are caused by these processes: Borna disease (BD) in mammals [15] and proventricular dilatation disease in birds [8]. The mammalian BDVs were the first to gain attention. They represent a homogenous group of viruses with strong genetical restriction and differ from the avian bornaviruses, which exhibit a broader sequence variation and do not replicate efficiently in mammalian cells [8]. Fatal neurological disorders in horses and sheep of unknown origin were observed in certain areas of Germany in the 19^th^ and 20^th^ centuries [15], [16]. The viral etiology was proved by Zwick in the 1920s [15], [17]. Nonpurulent meningoencephalitis with marked perivascular cuffing is the typical pathohistological presentation of BD [18]. BDV never caused epidemics although cases were observed more frequently in some years than in others [17], [19]. The disease is endemic in certain regions of Germany, the Swiss-Liechtenstein-Austrian Rhine valley and valleys of tributaries of the upper Rhine [19]. The only exception so far was a BD case in a pony in Styria (Austria) from which a new subtype of BDV was isolated [20], [21]. Very rarely other animal species such as rabbits, cattle, dogs [22] and zoo animals like new world camelids have been reported to succumb to the disease (for overview see [19]). The possibility of infections of humans with BDV was hypothesized over years based on antibody findings and later putative BDV sequences obtained from human specimens, but this has been questioned recently since reported human-derived BDV sequences proved to be inadvertent laboratory artefacts [23], [24] and psychiatric syndromes could not be linked to BDV antibodies [25]; moreover the specificity of antibodies against BDV in humans remains questionable since they have been found to be more frequent in patient groups with chronic diseases [26], [27] which points towards a high percentage of immunological cross-reactivity not linked specifically with BDV. Within the geographical distribution range of the disease some locations are more frequently affected than others [28], [29]. It often occurred in the past that one farm had single losses due to BD over years but neighboring farms had no losses at all, which raised several speculations about the origin of the disease [29]. The disease is more frequent in spring and early summer and has a seasonal low in autumn [19]. The incidence of BD decreased in the 1970s and has remained at a low level since [19]. Experimental infections of rodents support a possible reservoir function by persistent tolerated infections in some animals following different modes of infection [30], [31] and a spreading of the virus via urine [32]. However, a natural reservoir in rodents was not found [33], [34]. The epidemiological pattern of BD as well as the geographic clustering indicates an existence of reservoir species for BDV [19]. Thus it was a major breakthrough when BDV infections in bicolored white-toothed shrews (*Crocidura leucodon)* were reported from Switzerland [35], [36]. The sequences obtained from the shrews fit into the geographical cluster of the Swiss-Liechtenstein-Austrian-Rhine valley group. The shrews had been trapped near locations where BD in horses had been reported in the years before.

To further elucidate the role of shrews in natural BDV infection the objectives of this study were to investigate (i) bicolored white-toothed shrews from endemic regions in Germany, (ii) bicolored white-toothed shrews from locations where BD had not been reported during the last decades, and (iii) other species of shrews. The role of shrews as a natural reservoir of BDV would be further supported if infections were demonstrated in shrews of another, geographically separated endemic area which harbor BDVs that correspond genetically to the area.

## Results

### Sample Collection and Shrew Identification

**Figure 1:**
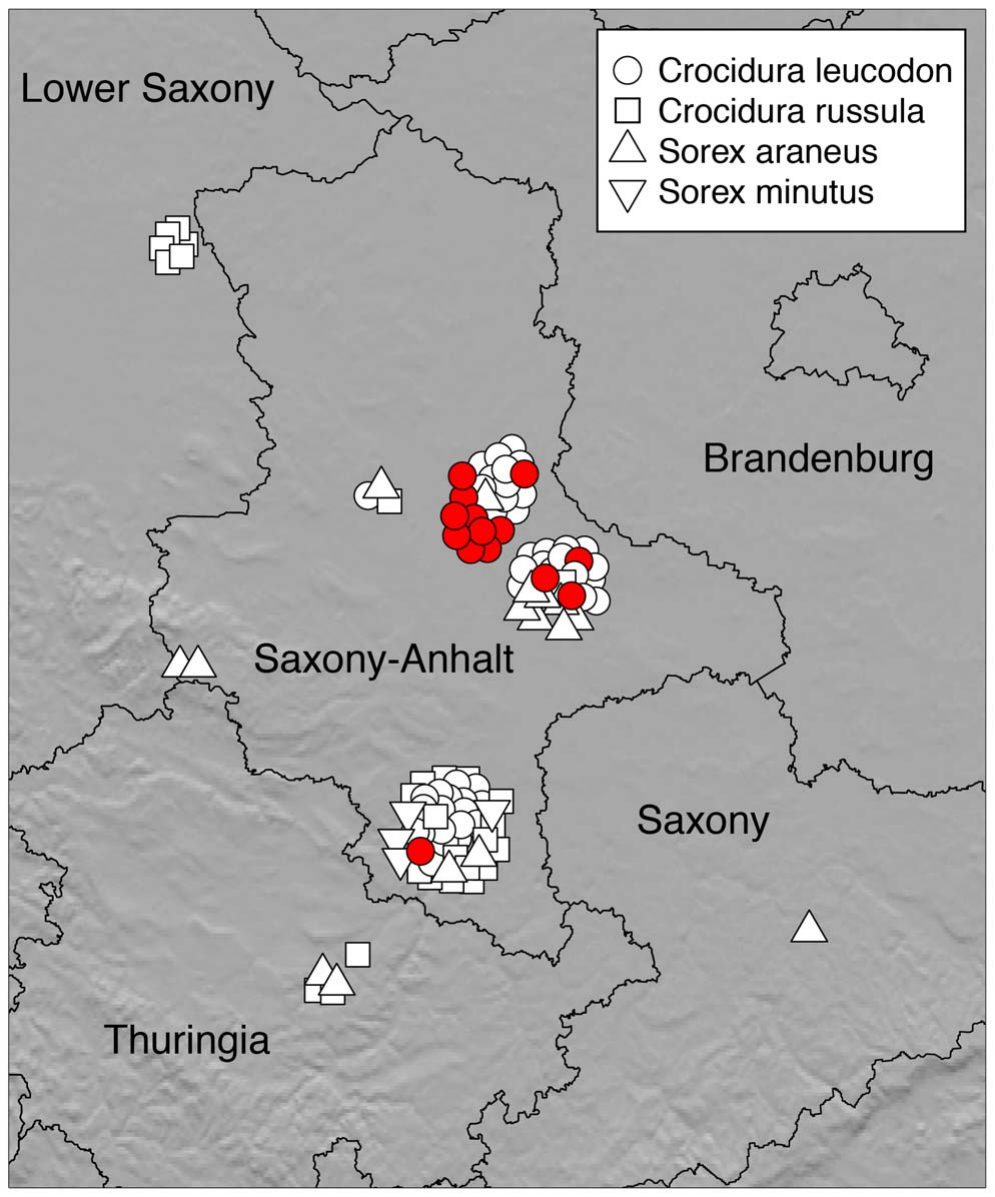
Collection sites of shrews and locations where BDV-positive white-toothed bicolored shrews were found. White symbols, specimens collected; red circles, BDV-positive *C. leucodon*.

From September 2005 to December 2010 a total of 107 shrews were collected (Table 1, Figure 1). The collection comprised 58 individuals of the species *Crocidura leucodon* (HERMANN 1780), 28 individuals of the species *Crocidura russula* (HERMANN 1780), 16 individuals of the species *Sorex araneus* (LINNAEUS 1758), four individuals of the species *Sorex minutus* (LINNAEUS 1766), and one individuum of the species *Sorex alpinus* (SCHINZ 1837). Body weight and size fit to the parameters expected for the corresponding species (Table S1, S Figure S1).

**Table 1:** Overview of shrew collection and individuals found to be positive for BDV.

Species	Country	Federal State	Location of positives^a^	Year	Number tested	Number positive^b^
*Crocidura russula*	Germany	Lower Saxony	–	2008	1	0
				2009	5	0
		Saxony-Anhalt	–	2005	1	0
				2006	1	0
				2007	4	0
				2008	11	0
				2009	1	0
				2010	2	0
		Thuringia	–	2005	2	0
*Crocidura leucodon*	Germany	Saxony-Anhalt	Buhlendorf	2005	2	0
			Freyburg/U.	2006	10	3/2/1
			Güterglück	2007	28	3/2/2
			Roβlau	2008	10	4/4/0
				2009	7	4/4/0
				2010	1	0
*Sorex araneus*	Germany	Saxony-Anhalt	–	2005	1	0
				2006	1	0
				2007	5	0
				2008	4	0
				2009	2	0
		Saxony	–	2006	1	0
		Thuringia	–	2007	1	0
				2009	1	0
*Sorex minutus*	Germany	Saxony-Anhalt	–	2007	1	0
				2008	1	0
				2009	2	0
*Sorex alpinus*	Italy	South Tyrol	–	2006	1	0
*5*	2	5	4	2005–2010	107	14

a not allocated to the year (Buhlendorf CL62 found in 2008, Freyburg/U. CL64 found in 2007, Roβlau CL19 found in 2006, CL77 and CL78 found in 2009, for Güterglück see Tab. 2);

b positive: BDV RNA (RT-PCR)/BDV antigen (immunohistochemistry)/nucleoprotein element genome integration (PCR without RT).

**Figure 2:**
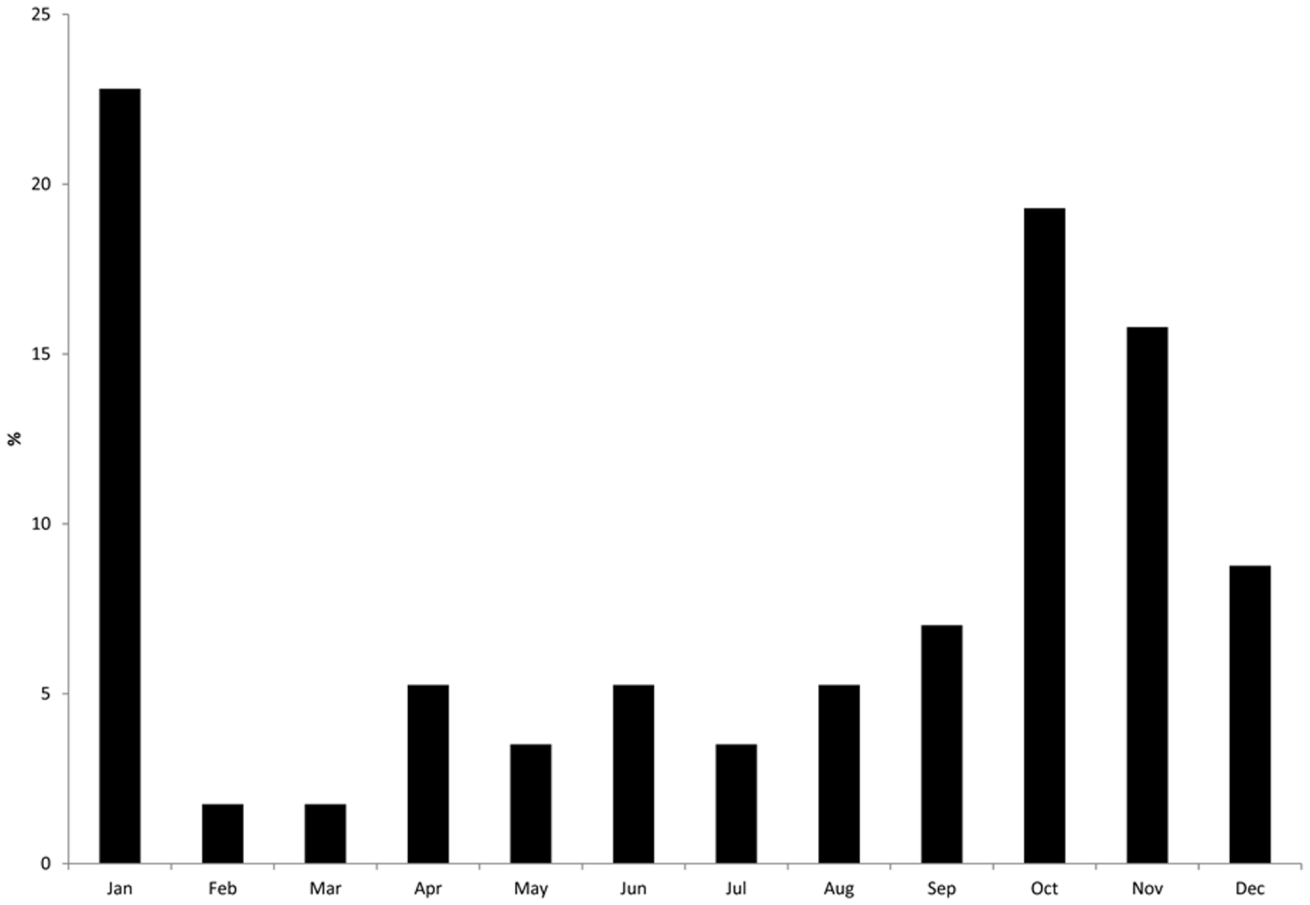
Relative trapping frequency (%) of the bicolored white-toothed shrews collected in this study (2005–2010). Number of shrews: 57 (from one shrew no exact collection date was available).

Bicolored white-toothed shrews were collected all year round but most frequently in autumn and early winter (Figure 2).

### Investigation by BDV RT-PCR, BDV (DNA) PCR and Cytochrome b Gene PCR

**Figure 3:**
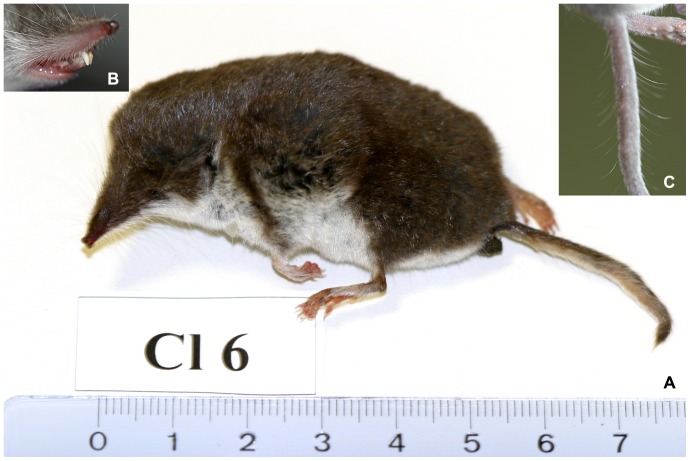
Bicolored white-toothed shrew (*C. leucodon,* HERMANN 1780) and its taxonomic identification. A. Bicolored body. B. White teeth of *Crocidura* species. C. Eyelashes-like hairs on tail of *Crocidura* species.

14 out of 107 shrews proved positive for BDV nucleic acid by RT-PCR. All positive individuals (CL17, CL18, CL19, CL35, CL54, CL62, CL64, CL72, CL73, CL74, CL75, CL76, CL77, CL78) belonged to the species *C. leucodon* (Figure 3) and were collected at three different locations in central Saxony-Anhalt and one location in the southern part of Saxony-Anhalt (Figure 1). Thus 14 out of 58 (24%) bicolored white-toothed shrews from Saxony-Anhalt were positive for BDV. All other shrew species were negative for BDV nucleic acid (Table 1). At one property in central Saxony-Anhalt, Güterglück, shrews were collected over five years. Here, nine of 17 bicolored white-toothed shrews (53%) were positive in four consecutive years (Table 2).

**Table 2:** BDV infection dynamics in bicolored white-toothed shrews (*Crocidura leucodon*) in one collection area (one property in Güterglück, Saxony-Anhalt, Germany) over a five year period.

Year	2005	2006	2007^a^	2008^a^	2009	Total
Total	1	2	8	3	3	17
Number of positives	0	2	2	3	2	9
Designation of positives	–	CL17, CL18	CL35, CL54	CL72, CL73, CL74	CL75, CL76	–
Percentage of positives	0%	100%	25%	100%	67%	53%

a three other individuals of *C. leucodon* (CL21, CL58, CL60) collected in 2007/2008 at two other properties in Güterglück where horses were kept were negative.

From selected BDV-positive shrews (CL17 and CL18) genetic material was amplified in all organs investigated (brain, heart, lung, liver, kidney, spleen, stomach, intestine, urinary bladder) indicating a wide distribution of the virus within the body of infected animals. Shrews CL19 and CL35 reacted differently: BDV genetic material was amplified in the brain but not in the other organs with two exceptions: stomach, lung (Table S2).

In addition, other insectivores, including a mole and a hedgehog, collected in BD-endemic areas of Saxony-Anhalt, were negative for BDV. Earthworms from the property with BDV infections in shrews over years (Güterglück, Saxony-Anhalt, Germany) were negative for BDV nucleic acid.

BDV DNA [by employing BDV PCR without reverse transcription (RT) step] was demonstrated in three shrews (CL17, CL54, CL64). Only the BDV primer pair established by Sorg and Metzler [37] within the nucleoprotein (N) gene detected BDV DNA [(242) 5′-GTCACGGCGCGATATGTTTC-3′ (261), (508) 5′-GATGACGATCCTATCACAACC-3′ (491), 267 bp, numbering corresponds to GenBank accession number U04608]. The nucleotide sequences obtained by the BDV DNA PCR were identical to those achieved by RT-PCR for the corresponding individual. All other primer pairs used for amplification of BDV [other parts of the N gene and the phosphoprotein (P) gene] did not detect BDV DNA.

The shrew species was confirmed by cytochrome b PCR.

### Sequencing and Phylogenetic Analysis

The BDV sequences determined in this study (1824 bp, consisting of complete N, P, and X genes) were deposited in GenBank database under the following accession numbers: EU622878 (CL17), EU622879 (CL18), KJ127543 (CL19), KJ127544 (CL35), KF724700 (CL54), KF724701 (CL62), KF724702 (CL64), KF724703 (CL72), KF724704 (CL73), KF724705 (CL74), KF724706 (CL75), KF724707 (CL76), KF724708 (CL77), and KF724709 (CL78).

The partial (505 bp) sequences of *C. leucodon* cytochrome b gene were deposited in GenBank under the following accession numbers: EU622880 (CL17), EU622881 (CL18), KJ147145 (CL19), KJ147146 (CL35), KF724690 (CL54), KF724691 (CL62), KF724692 (CL64), KF724693 (CL72), KF724694 (CL73), KF724695 (CL74), KF724696 (CL75), KF724697 (CL76), KF724698 (CL77), and KF724699 (CL78).

**Figure 4:**
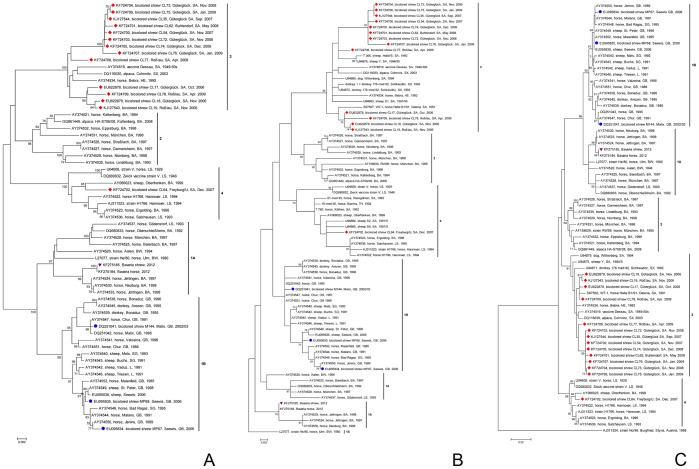
Phylogenetic analysis of BDV sequences obtained from bicolored white-toothed shrews. A. 1824(P), C. 1110 bp nucleic acid sequences within the gene coding for p40 (N) of 14 BDVs obtained from naturally infected bicolored white-toothed shrews from Germany determined in this study (red diamonds), three Swiss [35], [36] (blue circles) and one Bavarian [39] (purple triangle) *Crocidura leucodon*-derived BDVs, and other representative BDVs mainly from spill-over hosts, which succumbed to BD. Evolutionary analyses were conducted in MEGA5 [72]. For tree 4A (unrooted tree, 63 nucleotide sequences) the Neighbor-Joining method was used. For trees 4B (76 nucleotide sequences, outgroup BDV He/80, oldest available BDV sequence of the region where BDV was first reported) and 4C (69 nucleotide sequences, outgroup BDV No/98, most distant mammalian BDV known so far originating from a location far away from the BDV endemic regions) the Maximum Likelihood method was performed. The percentage of replicates in the bootstrap test (1000 replicates) is shown next to the branches. Values less than 70% are hidden. Cluster 1: Southwest Germany and Southern Rhine valley group, 1A. Baden-Wurttemberg and Bavaria west of Augsburg (Swabia) as well as central Bavaria around Munich, Germany, 1B. Graubuenden and Sankt Gallen, Switzerland, The Principality of Liechtenstein, and Austria (south of Lake Constance); Cluster 2: South German group (major parts of Bavaria), 3. Southern Saxony-Anhalt and Saxony (mainly along and between rivers Saale and Elbe south of Magdeburg); Cluster 4: Central German group: Lower Saxony, northern Saxony-Anhalt, Thuringia and bordering Saxony-Anhalt (mainly west of the river Saale), northern parts of Bavaria, Germany. BW, Baden-Wurttemberg; HE, Hesse; LS, Lower Saxony; SA, Saxony-Anhalt; SX, Saxony; TH, Thuringia (Germany); GB, Graubuenden; SG, Sankt Gallen (Switzerland); L, Liechtenstein (The Principality of Liechtenstein).

BDV sequences obtained from shrews cluster corresponding to the territory (Figures 4A, 4B, 4C, Figures S2, S3).

The sequences of the shrews CL17, CL18, CL19, and CL78 collected in Güterglück and Roβlau in 2006 and 2009 are related to the BDV sequence of WT-1 [38] derived in 1991 from a horse with BD in Dalena [28], a village located 39 kilometers southwest of these towns (Figure 4C). BDV sequences of horses 116 med 91 and 191 med 91 cluster close to them (GenBank accession numbers KJ494662 and KJ494663, Figure S3). These horses succumbed to the disease in 1991 in the villages of Hundeluft and Gübs [28] which are 47 kilometers apart. Güterglück lies about half way between them, with Roβlau 21 km southeast of Güterglück.

The BDV sequences obtained during the following years (2007–2009) from shrews collected at the same property in Güterglück (CL35, CL54, CL72–76) form a similar cluster and are closely related to the sequence found in a shrew collected near Buhlendorf, 6 kilometers from Güterglück, in 2008 (CL62).

The other BDV sequences from shrews collected in Roβlau (CL19, CL77) fit to the region.

**Figure 5:**
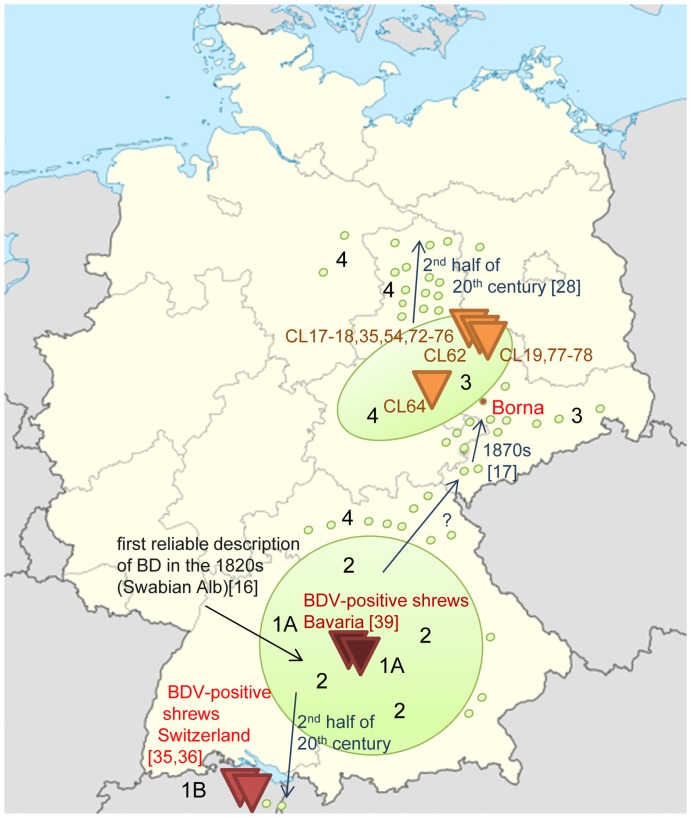
Locations of BDV-positive bicolored white-toothed shrews within BD endemic regions. Endemic regions of BD (green circles, main endemic areas; green dots, BD occurs but is rare), locations at which positive bicolored white-toothed shrews were found in this study (orange triangles) and were reported in Switzerland [35], [36] (red triangles) and Bavaria [39] (brown triangles), genetic cluster groups (1A,B; 2; 3; 4), and movements of BD into new territories (blue arrows); BD was first described by Authenrieth in the Swabian Alb [16]; there was never an epidemic in the city of Borna but the disease occurred frequently in horses on farms in the district of Borna from the late 1880s until the 1960s but it is rare there now [17]; BDV-positive shrews from three major clusters (1, 3, 4) have been found so far. The distribution of BD in the scheme is based on reference data [16], [24], [28], [48], [55], [61], [75]–[81] and report data from Berichte über das Veterinärwesen im Königreich(e)/Freistaat Sachsen – for details see reference [17] page 23, references 285 and 286, and page 24, references 309 and 310.

The BDV sequence of the bicolored white-toothed shrew found in Freyburg/Unstrut (CL64) belongs genetically into sequence group 4 (Figures 4, 5). It is closely related to a BDV sequence of a horse which had BD 16 years and eight months before the shrew was found. The horse was kept in Borau-Kleben near Weiβenfels [28] which is situated 17 km east of Freyburg/Unstrut (Figure S3).

The BDV sequences of the shrews from the property in Güterglück in 2006 differed slightly to that of the following years (98% identity) but from 2007–2009 all BDV sequences of the shrews from this property were almost identical (99% identity, see Table S3).

### Immunohistochemical Investigations

**Figure 6:**
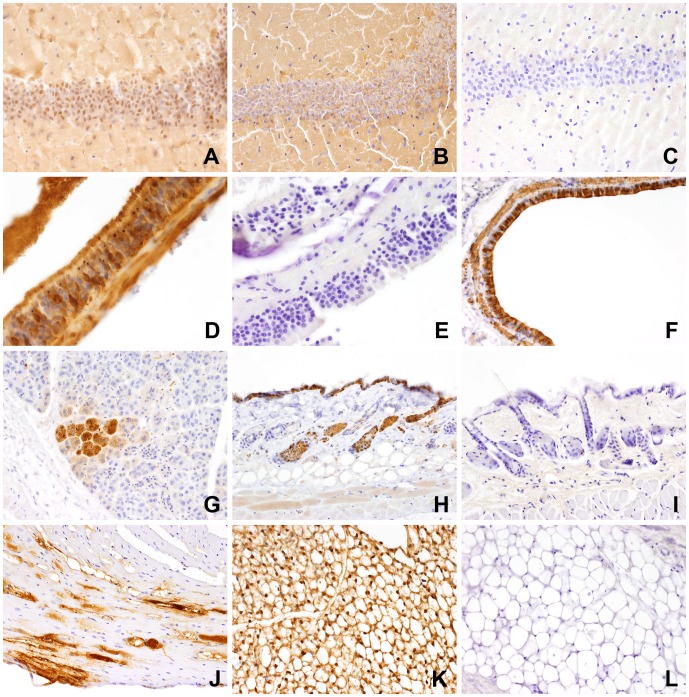
Immunohistochemical staining patterns of different organs with the polyclonal anti-P antibody and the monoclonal antibody Bo18. A. Diffuse immunostaining in the area of the dentate gyrus, brain, anti-P antibody; shrew CL72; B. Diffuse immunostaining in the area of the dentate gyrus, brain, Bo18 Ab; Note slightly different immunostaining with presence of nuclear signals with anti-P antibody and absence of nuclear signals with Bo18 Ab. C. Absence of immunostaining in the dentate gyrus of a BDV-negative shrew; brain, CL61; D. Abundant immunostaining of olfactory epithelium with presence of strongly stained intranuclear bodies, anti-P antibody, shrew CL73; E. Lack of immunostaining in the olfactory epithelium of a BDV-negative animal, anti-P antibody, shrew CL61; F. Immunostaining of entire bronchial epithelium and underlying smooth muscle layer, lung, anti-P antibody, shrew CL62; G. Immunostaining of a group of glandular epithelial cells, salivary gland, anti-P antibody, shrew CL62; H. Immunostaining of keratinocytes and epithelia of hair follicles, skin; shrew CL62; I. Complete absence of immunostaining in the skin of a BDV-negative animal; anti-P antibody, shrew CL61; J. Immunostaining of myocardial cells; heart; Bo18 Ab; shrew CL76; K. Abundant immunostaining of adipocytes in abdominal fat tissue; anti-P antibody; shrew CL73; L. Complete absence of immunostaining in the fat tissue of a BDV-negative animal; anti-P antibody.

Twelve RT-PCR-positive shrews showed abundant immunostaining. Brain and spinal cord had no infiltration of inflammatory cells and had a diffuse pattern of specific immunoreactivity indicating that all components of central nervous system tissue (neurons, glial cells and their processes) harbored BDV proteins (Figures 6A, 6B). Sometimes the staining intensity was more pronounced within neuronal nuclei and axons. Also sciatic nerves and other peripheral nerves visible in skeletal muscle samples and other tissues were strongly positive. Here the positive signals were strongest within the axons and less distinct within myelin sheaths. Vegetative nerve fibers of different diameters and occasionally vegetative ganglia were clearly positive. Labelled vegetative ganglia were consistently present within the muscle layers of the intestine. Nerve fibers were positive in the dermis and subcutis, in interstitial tissue of salivary glands, pancreas, heart, kidney, liver and lung, in the muscularis, submucosa and propria of the gastrointestinal tract, and in the red pulp of the spleen. A large variety of other tissues were also strongly immunopositive: olfactory epithelium (Figure 6D), bronchial epithelium (Figure 6F), epithelia of salivary glands (Figure 6G), exocrine pancreas, oral cavity including tongue, as well as keratinocytes and hair follicle epithelia of the skin (Figure 6H). Parenchymal cells of the large abdominal organs (liver, kidney) were predominantly negative, with the exception of single hepatocytes. Among mesenchymal tissues, immunostaining was widely positive in smooth muscle cells (gastrointestinal tract, skin, blood vessel walls, bronch(iol)i (Figure 6F), myocardium (Figure 6J), skeletal muscle and fat tissue (Figure 6K). Both antibodies produced comparable staining patterns and intensities. The anti-P-antibody tended to produce a more distinct nuclear staining pattern, while the staining achieved with the Bo18 antibody was more diffuse. The negative controls did not show any immunohistochemical signals (Figures 6C, 6E, 6I, 6L). Two bicolored white-toothed shrews (CL19, CL35) displayed no positive signals in immunohistochemistry despite positivity in BDV RT-PCR.

### Statistical Investigations

The statistical analysis of BDV positive individuals within shrew species revealed significant differences between *C. leucodon* and *C. russula* (p = 0.005, Mann-Whitney-U-test 2-tailed) and *C. leucodon* and *S. araneus* (p = 0.030, Mann-Whitney-U-test 2-tailed). Differences between *C. leucodon* and *S. minutus*/*S. alpinus* were not investigated due to the low number of specimens collected.

## Discussion

Bicolored white-toothed shrews *(Crocidura leucodon,* HERMANN 1780) are indigenous hosts of BDV because: (i) direct BDV signals were not found in other shrew and rodent species, (ii) bicolored white-toothed shrews display no tissue damage despite BDV infection, (iii) they are distributed within BD endemic territories and their distribution fits to the elevations and to the northern fringe of BD occurrence, (iv) the sequences obtained from infected shrews fit exactly into the cluster of geographic origin that have been described for mammalian BDV of horses and sheep within the different endemic regions, and (v) the solitary, mating, and dispersal behavior of shrews is different from that of rodents and corresponds to the special features of BD and the formation of epidemiological hot spots; the relevance of the identification of a native virus reservoir is supported by the following: (vi) BDV infections are maintained in local shrew populations, (vii) the yearly varying and seasonal incidences of BD correlate well with the population dynamics of a species with high metabolic rates strongly depending on a constant food supply, and (viii) BDV N elements can be integrated into the genome of bicolored white-toothed shrews after infection.

### (i) Bicolored White-toothed Shrews are the only Reservoir Species of Mammalian BDV Identified so Far

The wide distribution of endogenous BDV N elements and the results of numerous experimental infections indicate that many species can be infected by BDV. Despite this, natural BDV infections are rarely reported in mammals.

In this study we focused on shrews because all investigations of more than 500 wild rodents of at least ten species from BD endemic regions done in the past revealed all of them to be negative (for overview see Table 3). These results and the wide distribution of rodents, which is in contrast to the restricted occurrence of BD, indicated that other species than rodents with special features are more likely to be a reservoir of BDV. After the detection of BDV in bicolored white-toothed shrews the investigation of further shrew species became important. Bicolored white-toothed shrews were BDV-positive at 4 of 9 locations investigated in this study. BDV-positive bicolored white-toothed shrews were found at all locations with recent cases of BD in horses [35], [36], [39] (see Table 3).

**Table 3:** Overview of potential reservoir species investigated in BD endemic regions so far.

Species	Region	From or nearfarms with BD	From locationswithout BD	Numbertested	Numberpositive	Reference
*Rattus norvegicus*	Bavaria	yes	no	115	0	RD^a^
*Rattus rattus*	Bavaria	yes	no	3	0	RD^a^
*Mus musculus*	Saxony-Anhalt	yes	no	175	0^b^	[33]
	Bavaria	yes	no	15	0	RD^a^
	Bavaria	yes	no	?^c^	0	[34]
	Switzerland	yes	no	7	0	[36]
	Bavaria	yes	no	28	0	[39]
*Apodemus sylvaticus*	Bavaria	yes	no	10	0	[39]
*Apodemus flavicollis*	Bavaria	yes	no	2	0	[39]
*Apodemus sp.*	Switzerland	yes	no	46	0	[36]
*Micromys minutus*	Bavaria	yes	no	2	0	[39]
*Microtus sp.*	Switzerland	yes	no	5	0	[36]
	Bavaria	yes	no	41	0	[39]
*Myodes glareolus*	Bavaria	yes	no	2	0	[39]
*Arvicola terrestris*	Switzerland	yes	no	2	0	[36]
	Bavaria	yes	no	9	0	[39]
*Clethrionomys glareolus*	Switzerland	yes	no	9	0	[36]
Mice not classified	Switzerland	yes	no	87	0	[35]
*Talpa europaea*	Switzerland	yes	no	8	0	[35]
	Saxony-Anhalt	no	yes	1	0	tp
*Erinaceus europaeus*	Saxony-Anhalt	no	yes	1	0	tp
*Sorex minutus*	Saxony-Anhalt	no	yes	4	0	tp
*Sorex araneus*	Switzerland	yes	no	6	0	[36]
	Bavaria	yes	no	5	0	[39]
	Saxony-Anhalt	no	yes	13	0	tp
	Saxony	no	yes	1	0	tp
	Thuringia	no	yes	2	0	tp
*Crocidura russula*	Bavaria	yes	no	1	0	[39]
	Lower Saxony	no	yes	6	0	tp
	Saxony-Anhalt	no	yes	20	0	tp
	Thuringia	no	yes	2	0	tp
*Crocidura leucodon*	Switzerland	yes	no	3	3	[35]
	Switzerland	yes	no	6	2	[36]
	Bavaria	yes	no	20	2	[39]
	Saxony-Anhalt	no	yes	58	14	tp

a specimens were collected in the district of Feuchtwangen, Central Franconia, Bavaria, Germany 1994–1995 and investigated by Ralf Dürrwald, the results were not published but mentioned in reference [19];

b tested by intracerebral infection of rabbits;

c number not mentioned in the publication; tp, this paper; in total at least 558 rodents of at least ten species, nine moles, one hedgehog, 27 common shrews, 29 greater white-toothed shrews, four Eurasian pygmy shrews from BD endemic regions have been investigated for BDV so far, all were negative; 21 of 87 bicolored white-toothed shrews were positive for BDV; at all locations with BD in the recent past BDV-positive bicolored white-toothed shrews were found; at 4 of 9 locations without BD cases in the recent past BDV-positive bicolored white-toothed shrews were detected; only investigations in the regions of classical Borna disease were considered in this table (lowland regions of Baden-Wurttemberg, Bavaria, Lower Saxony, Saxony-Anhalt, Saxony, Thuringia in Germany and the upper Rhine valley and valleys of tributaries of the upper Rhine in Switzerland, The Principality of Liechtenstein and Austria).

The finding that bicolored white-toothed shrews are infected naturally does not rule out the existence of other reservoir species. Recently antibodies against BDV have been detected in a few wild rodents in Scandinavia [40] and Bavaria [39], but as long as the controversially discussed specificity of BDV antibodies has not been clarified discussions about BDV infections in these species remain speculative (antibodies against BDV have been found worldwide in almost all species investigated). So far no BDV sequences are available which prove the existence of BDV in bank voles in Finland [40].

### (ii) BDV Infects Shrews but Causes No Tissue Damage

First hints indicate that BDV might be very well tolerated by shrews. There are no tissue alterations which distinguish infected from non-infected animals, neither in the nervous system nor in other organs. The distribution pattern of BDV proteins N and P observed immunohistochemically in the shrews of our study resembles the viral distribution of neonatally infected immunotolerant animals with virus replication in a large variety of tissues [41]–[44]. Very remarkable is the completely diffuse viral distribution in the central nervous system and the absence of an inflammatory reaction. In contrast to neonatally infected Lewis rats, which show neuroanatomical abnormalities like cerebellar atrophy and dentate gyrus degeneration [45], there are no noticeable neuroanatomical alterations in the brain of shrews. It is generally known from the experimental rodent model that BDV infection can be transmitted via urine and contact [32] but also vertically by intrauterine transmission [46]. Shedding of virus, at highest titers in the urine, has been demonstrated in immunotolerant but never in immunocompetent animals. Thus it has been speculated that only animals infected in utero or as newborns may serve as natural vector due to their capacity of virus excretion.

An unexpected phenomenon in the shrews was the presence of large amounts of viral antigen in epithelial cells of the oral cavity and in keratinocytes of the skin. Skin had not been investigated for viral presence in previous experimental studies on BDV distribution and shedding. In shrews the presence of virus in the skin could be of considerable epidemiological relevance and a source of infectious virus for natural (obviously shrews) and spill-over hosts (such as horses, sheep and other susceptible animals).

Shrews CL19 and CL35 were negative in immunohistochemistry (including brain and spinal cord). They differed from the other BDV-infected bicolored white-toothed shrews by the restriction of BDV nucleic acid to the brain. The immune status of these shrews is unknown. They could represent animals at an early stage of infection. Due to the collection time late in the year it is more likely that they reflect immunocompetent individuals which were able to prevent or clear infection outside the brain. Traces of BDV signals in stomach und lung of these shrews indicate that virus was still present in their environment.

### (iii) Geographic Distribution of Bicolored White-toothed Shrews Corresponds to BD Epidemiology

BDV was detected in bicolored white-toothed shrews in BD endemic regions at locations with recent BD cases in spill-over hosts [35], [36], [39] and without [this paper].

Looking at maps, the main regions of occurrence of classical BD are lowland areas bordered by low mountain ranges (compare with Figure 5). Valleys and gaps between low mountain ranges appear to have enabled the further distribution of BD. No mountain border exists east of the East German endemic region, but BD does not occur there.

Bicolored white-toothed shrews are widely distributed in central Europe [47]. They are usually found below 1000 m in elevation except in the Alps, where they may be found as high as 1600 m [35]. These facts fit to the features of BD which has been reported to occur at elevations lower than 700 meters above sea level in Germany [48] and at higher elevations in the Alps [35]. The northern distribution range of this species in central Europe is restricted to a line from Oldenburg, Bremen over the district of Salzwedel to Potsdam, southern Berlin (in Germany) and to Warsaw and Bialystok (in Poland) [49]. Investigations by Martens and Gillandt in 1978/79 [50] and by Weber in 1983 [51] revealed a further extension of the northern distribution of *C. leucodon* during the last decades. Whereas this northern distribution border fits well to the occurrence of BD in endemic regions, it is the limited distribution of BD itself which is in contrast to the wide distribution of bicolored white-toothed shrews in Europe, especially in western and eastern Europe, where no clearly confirmed BD cases have been reported so far. In western Europe *C. leucodon* is distributed in the eastern parts of France and in eastern Europe it populates habitats of the countries of the Balkan peninsula, Poland and Ukraine [52]. Investigations by Richter [49] in the eastern part of Germany have shown that the bicolored white-toothed shrew is more common in regions of the lowlands of Thuringia and Saxony-Anhalt. These regions fit exactly to the distribution of BD in horses and sheep (especially in eastern Germany the maps of distribution of BD - shown by Dürrwald, see Figure 2 of reference [28] - fit exactly to the distribution frequencies of *C. leucodon* - data by Richter, see Figure 5 of reference [49]). In other regions especially in northwestern Germany *C. russula* is more common and BD is rare. *C. russula* (greater white-toothed shrew) is mainly distributed westwards of the Rhine and southwards of the Alps, but occurs also between Rhine and Elbe together with *C. leucodon*. *C. suaveolens* (lesser white-toothed shrew) is widely distributed in the eastern part of Europe and not competing with *C. leucodon* in the endemic areas of BD in Thuringia and Saxony-Anhalt, but well-distributed in Bavaria, where 3 *Crocidura* species exist [53], [54]. Regions of Bavaria in which BD occurs fit to the distribution area of *C. leucodon* [55]. Mean annual precipitation lower than 900 mm, mean annual temperatures of 8°C, elevation lower than 350 m, low forest cover and urban environment were determined to be optimal habitats for *C. leucodon* [56]. In 2012 two BDV positive bicolored white-toothed shrews were found in farms in Bavaria with recent cases of BD in horses [39]. BDV sequences from shrew and horse of the same stable were identical indicating transmission between both species [39]. Despite the wider distribution of *C. leucodon* compared to the BD endemic areas the population density of this species may play a role in virus transmission. Shrews found in southern Saxony-Anhalt were dominated by *C. russula* (this study) which may explain the low detection rate of BDV.

Two points could explain the natural infection of bicolored white-toothed shrews within a limited area of their normal territory. Either a more recent evolutionary event introduced BDV to this species, or there is another infection source within the BD endemic regions linked to *C. leucodon* but not to other shrew species. The strong genome conservation of mammalian BDV [38] could be caused by the involvement of another species in the transmission cycle due to the restrictions that are put genetically on viruses which jump between far distant host species [57]–[59]. In a first attempt to investigate potential food of shrews for BDV we collected earth worms from the property where BDV-infected shrews had been found over four consecutive years. All earth worms were negative for BDV nucleic acid.

### (iv) BDV Sequences Obtained from Shrews Cluster Correlating to the Geographic Region

The BDV sequences of bicolored white-toothed shrews fit well into the group of mammalian BDVs and are unconnected to avian bornaviruses. The single description of mammalian BDVs in birds [60] has to be viewed critically because the sequences obtained resemble those of laboratory strains [19]. In addition to the description of mammalian BDV sequence groups by Kolodziejek *et al.* [61] the integration of sequences from BDVs of shrews allows the definition of major groups (for definition see Figure 4, for geographic distribution see Figure 5). Local BDV sequences remain stable for decades [61]. This allows conclusions about the origin of BDVs and relationships between the sequence groups. BD was comprehensively reported near the Swabian Alb as early as the early 19^th^ century [16]. Sequence group 1A is located in this region (Figure 5) and displays a high variability (Figure 4B). In Bavaria viruses of three sequence groups are prevalent (1A, 2, 4). Group 1B is bound to the Rhine valley and valleys of the tributaries of the Rhine in Switzerland, The Principality of Liechtenstein and Austria. Sequence group 3 is located in the region around the city of Borna. Group 4 is the most northerly.

Outliers (acc. nos. AY374534, AY374537 Figure 4, U95356, horse TREPTOW Figure S3) are most probably from horses and sheep which were infected in the region of the cluster and later on transported to nonendemic regions where they developed disease after a long incubation time. Shrews collected in Güterglück moved by themselves into the former chicken house and garage of the property. Shrews collected in Roβlau were caught by cats at different locations and brought to the collection site which may explain the higher BDV sequence variation.

### (v) Behavior Patterns of Shrews Fit to BD Hot Spot Formation

Shrews are insectivores [52]. Insectivore-like mammals appeared in the Late Cretaceous [62]. Typically shrews are solitary animals [52]. They rear up to four litters from March to September [63]. Natal dispersal is mainly observed in female weanlings [64]. Most of the offspring settles locally, which creates a high potential for inbreeding [65]. Dispersal distance is low [66]. This inbreeding and low distance dispersal feature could fit to the limited distribution of BDV within endemic territories.

In the case of the shrews from the village of Güterglück it is possible that the shrews trapped over the years reflect different family clans of which the two shrews of 2006 and the shrews of 2007–2009 were from BDV-infected ones. The accumulation of infected reservoir animals in human settlements can contribute to disease hot spot formations. Some epidemiological hot spots of BD have been known in the past. Such an epidemiological hot spot had been the Zoological Garden Erfurt where in the 1970s and 1980s several herbivore zoo animal species succumbed to BD [67]. Investigations by Netzer in one district of Bavaria in the early 1950s revealed that there were different hot spots of BD in horses not only within the district but also within the villages themselves; he found that BD in horses was more common in distinct parts of the villages and within these some neighboring farms were more often affected over the years than others [29]. The property in Güterglück, where BDV-positive bicolored white-toothed shrews were collected, represents a location where hot spot formation was not obvious by disease in spill-over hosts because there were no horses and sheep on-site. In the immediate neighborhood of this property chickens, ducks and pigs were kept in a small barn under poor hygienic conditions which may have attracted shrews. Investigations of epidemiological factors of BD revealed that BD more often occured in traditional farms where several different animal species were kept together and hygiene was poor [19], [28]. Güterglück is located in a lowland area between Magdeburg and Dessau in Saxony-Anhalt not far from the river Elbe. Although BD cases have been reported from this region in the past, BD has never been known in the village itself and the neighboring villages. The last BD cases in horses within this region were observed in the village of Nutha, 3 km from Güterglück, in 1958, and in the village of Leitzkau, 9 km away, in the early 1950s. According to local veterinarians, BD has not been observed in horses or sheep within that small area for at least 50 years.

### (vi) BDV is Maintained in Local Shrew Populations

Nine of 17 shrews collected over five consecutive years at one property from 2005–2009 were BDV-positive (see Table 2). The percentage of positives differed by the years. This feature fits with the yearly varying peaks that have been reported for BD [19]. Although the number of BDV-positive shrews was low in general (24%), the percentage of positives at the property in Güterglück was high (53%). Since BD has never been recorded in Güterglück this could support the hypothesis of a self-sustaining infection cycle of BDV in bicolored white-toothed shrews.

### (vii) Yearly Varying and Seasonal Incidences of BD Correlate Well with the Population Dynamics of Shrews

There has been a remarkable decrease of BD within the last decades, probably indirectly caused by modern agriculture and the resulting restriction of habitats and decreases in food resources of a supposed reservoir of BDV [19]. High metabolic rates make shrew populations vulnerable to alterations in food supply [52]. The yearly varying peaks of BD could reflect population dynamics of reservoir species.

Our study only involved shrews that were found/trapped by accident. The bicolored white-toothed shrews were more often trapped in autumn and early winter which indicates higher contacts between shrews and human settlements in the second half of the year. In this time shrews visit stables and houses. At first glance, this contrasts with the absolute frequency of cases of BD in horses and sheep which peaks in May/June but displays a nadir in September/October/November [19]. The natural incubation period of BD is unknown. Only a few experimental infections of horses have been conducted so far, but based on artificial intracerebral infections. The incubation periods of these experiments ranged from two to several weeks [15], [68]. No conclusion on natural incubation times can be drawn from this. Data of animals which were transferred from BD endemic regions to nonendemic regions and which later developed BD (alpaca lent from Bavaria to Hesse [69], horse exported from Germany to the UK [70]) indicate that the natural incubation period may be more than 2–5 months. An incubation period of 5±3 months would be in agreement with higher contact frequencies of *C. leucodon* to stables from late fall to middle winter.

### (viii) BDV N Elements can be Integrated into the Genome of Bicolored White-toothed Shrews after Natural Infection

BDV N elements were identified by BDV DNA PCR in three bicolored white toothed shrews but not in the other eleven BDV positive shrews investigated. This confirms for the first time genome integrations associated with recent BDV infections in the natural host, and correlates with results of experimental infections of laboratory mice and newborn bank voles [9], [71]. According to results of Kinnunen *et al.* [71] BDV genome integration depends on infection dose and time after infection and is not detectable in all experimentally infected animals. It may also depend on the age of infected animals because all experimental studies conducted so far were based on newborn individuals [9], [71]. The fact that 14 bicolored white-toothed shrews were positive by RT-PCR but not all of them expressed antigen or integrated BDV N elements could be due to sampling at different times after infection.

## Conclusions

This study provides evidence that *C. leucodon*, the bicolored white-toothed shrew, can harbor BDV without tissue damage in territories endemic for BD, whose BDV sequences fit to the epidemiological sequence cluster of the corresponding region. At one location a high percentage of BDV-positive shrews was identified in four consecutive years without locally associated spill-over disease in horses and sheep; this is a novel finding which points towards a self-sustaining infection cycle in bicolored white-toothed shrews. BDV infections continue to exist in endemic regions although the disease in horses and sheep has significantly decreased in recent years and almost vanished in some regions. Further investigations of *C. leucodon* and other insectivorous species may help to increase our knowledge on the epidemiology of BDV.

## Materials and Methods

### Sample Collection and Shrew Identification

A flyer with pictures of shrews was circulated to interest groups such as veterinarians, farmers and breeders of small animals, expressing our interest in collecting dead shrews.

The collection focused mainly on a few locations in the central and southern territory of Saxony-Anhalt in Germany which reflects the distribution area of the BDV sequence groups 3 and 4 [defined in this paper, see Figures 4 and 5]. Moreover shrews found in Thuringia, Saxony, Lower Saxony (Germany) and South Tyrol (Italy) were included in the study. We focused only on shrews which were caught by cats, trapped inadvertently in rodent traps, or found dead. Immediately after collection the shrews were stored at −20°C and later at −80°C. The shrews were numbered according to the initials of their Latin species name (CR, CL, SA, SAR, SM) and the order of collection (No. 1–107). In addition, we investigated some other insectivores from the central Saxony-Anhalt (one mole *Talpa europaea* FISCHER 1817, one hedgehog *Erinaceus europaeus* FISCHER 1814). From one property on which BDV positive shrews were found over the years (Güterglück, Saxony-Anhalt, Germany) 20 earth worms *Lumbricus terrestris* (LINNAEUS 1758) were collected in order to investigate the possibility of BDV infection in the food of shrews.

All shrews were weighed and their size measured in a frozen state. Pictures were taken of each shrew shortly before dissection. The exact species was determined either by visual inspection or, if this was not certain, the skulls were analyzed after dissection. Shortly before dissection, the animals were thawed and different organ samples were taken for BDV reverse transcription polymerase chain reaction (RT-PCR) and immunohistochemical investigation. BDV-positive shrews were also investigated by BDV PCR (without RT) and by PCR on the cytochrome b gene to confirm the exact species.

Epidemiological parameters of interest were location and the date the shrews were collected. Information about the estimated population of shrews within the territory and about the occurrence of BD was gathered.

### Investigation by BDV RT-PCR, BDV (DNA) PCR and Cytochrome b Gene PCR

Nucleic acid extraction was conducted by employing the QIAamp Viral RNA Purification Kit (QIAGEN, CA, USA) according to the manufacturer’s instructions. This kit is designed to isolate total nucleic acid (viral RNA, viral and cellular DNA). All BDV RT-PCRs were carried out using a One Step RT-PCR Kit (QIAGEN, CA, USA) as described by Kolodziejek *et al.* [61]. A screening RT-PCR (available also as nested PCR) within the p40 gene, which proved to be best for detection of classical and highly variant BDV strains was conducted [37] (for sensitivity examination see [20]). In addition, a larger group of organs from BDV-positive individuals was investigated. All BDV-positive shrews were finally investigated by BDV RT-PCRs with six further BDV-specific primer pairs, as described by Kolodziejek *et al*. [61]. For investigation of potential genome integration of BDV all above primer pairs were used and PCRs without reverse transcription were performed. Furthermore, one genomic PCR for determination of the exact species was applied. For this purpose one primer pair amplifying a 549 bp fragment of the mitochondrial cytochrome b sequence of *Crocidura leucodon* shrews was designed with the help of the Primer Designer program (Scientific and Educational Software, version 3.0) and synthesized by Invitrogen (Life Technologies, UK). The primers consisted of the following sequences: (568) 5′- TTGCAGGAGTACACCTGTTATT -3′ (589) and (1116) 5′- GGTTTTCAACTATGCTTGTGAT -3′ (1095). The numbering corresponds to the *Crocidura leucodon* cytochrome b gene sequence of GenBank accession number DQ065609. The BDV (DNA) PCR and the cytochrome b PCR were performed by employing the Qiagen Fast Cycling PCR Kit (QIAGEN, CA, USA) according to the manufacturer’s instructions.

### Sequencing and Phylogenetic Analysis

For sequencing, all amplification products were purified using PCR Kleen Spin Columns (BIO-RAD, CA, USA) following the manufacturer’s protocol. Sequencing in both directions was carried out either by Microsynth (www.microsynth.ch) or by the ABI Prism 310 genetic analyzer (Perkin Elmer, CA, USA) automated sequencing system as described by Kolodziejek *et al.* [61]. The compiled nucleotide sequences were submitted to BLAST (http://www.ncbi.nlm.nih.gov/BLAST/) for comparison with all corresponding sequences deposited in gene bank databases.

Prior to phylogenetic analysis multiple sequence alignments were performed using BioEdit Sequence Alignment Editor Version 7.0.9.0 and verified by Clustal X program (version 1.8). Phylogenetic trees were constructed on A. 63 nucleotide sequences (1824 bp long comprising complete N, P, and X genes; Fig. 4A, unrooted tree, Neighbor-Joining method), B. 76 almost entire p24 (P) gene sequences where only the two last nucleotides were missing (604 bp corresponding to nt positions 1272–1875 of reference strain V, GenBank acc. No. U04608; the length is determined by the shortest sequence) with strain He/80 as outgroup (Fig. 4B, Maximum Likelihood method), C. 69 almost entire p40 (N) gene sequences where only the three last nucleotides were missing (1110 bp corresponding to nt positions 54–1163 of reference strain V, GenBank acc. No. U04608; the length is determined by the shortest sequence) using strain No/98 as outgroup (Fig. 4C, Maximum Likelihood method), D. 63 nucleotide sequences (1824 bp long comprising complete N, P, and X genes; Figure S2, radial tree, Neighbor-Joining method), and E. 88 nucleotide sequences of a stretch within the p40 (N) gene (399 bp corresponding to nt positions 312–710 of reference strain V, GenBank acc. No. U04608; Figure S3, unrooted tree, Neighbor-Joining method). Phylogenetic analyses were conducted using the MEGA5 program [72]. For each tree the evolutionary history was inferred using either the Neighbor-Joining or the Maximum Likelihood method. The evolutionary distances were computed using the Kimura 2-parameter model. The optimal trees with the highest percentages of replicates in the bootstrap test (1000 replicates) were chosen.

### Immunohistochemical Investigations

Brain, spinal cord, peripheral nerves, nasal mucosa, salivary glands, heart, lung, stomach, intestine, pancreas, liver, kidney, spleen, skeletal muscle, and skin from the RT-PCR positive shrews were analyzed by immunohistochemistry. Tissue samples were fixed in 10% neutral buffered formalin, embedded in paraffin wax and stained according to a previously described protocol [73] with two different antibodies: (i) the monoclonal antibody Bo18 which is directed against amino acids 19–27 of the nucleoprotein (N) of BDV [74] and (ii), a polyclonal antibody directed against the entire recombinant phosphoprotein (P) of BDV [7]. As negative controls, the same organs of three RT-PCR negative *C. leucodon* shrews were stained according to the same protocol.

### Statistical Investigations

The Mann-Whitney-U-test was performed using the programme SPSS 15.0.

## Supporting Information

Figure S1 Species identification of shrews investigated in the study. A. Common shrew (*Sorex araneus,* LINNAEUS 1758); the brown color at the flanks which divides the dark brown back from the grey vent, the red tipped teeth and the smooth tail are typical; B. Pygmy shrew (*Sorex minutus,* LINNAEUS 1766); the small size and weight (of this individuum 3.405 g) are striking; note the red tipped teeth and the missing lashes at the tail which are characteristics of the genus *Sorex*; C. Mountain shrew (*Sorex alpinus*, SCHINZ 1837); the dark color of the body and the long tail are conspicuous; red teeth and the smooth tail are characteristics of the *Sorex-*species; D1. Bicolored white-toothed shrew (*Crocidura leucodon*, HERMANN 1780); the bicolored body and the lashes at the tail are striking; the tail is short; members of the genus *Crocidura* do not possess red tipped teeth; D2: Bicolored white-toothed shrew (*Crocidura leucodon*, HERMANN 1780); another specimen; note the white color ventral, the white teeth and the lashes under the tail; E. Greater white-toothed shrew (*Crocidura russula*, HERMANN 1780); this species has a grey vent, the teeth are white, and there are lashes at the tail.
(TIF)

Figure S2 Radial phylogenetic tree representing the position of the BDV sequences obtained from shrews. Sequences of BDV-positive bicolored white-toothed shrews are marked with red diamonds [this paper], blue circles [35], [36] and purple triangle [39]. Sequences of BDVs from spill-over hosts do not have marks. The tree (Neighbor- Joining method) includes a 1824 bp stretch of the BDV genome comprising the N, P, and X genes and consists of 63 sequences. Bootstrap values less than 99% are hidden.
(TIF)

Figure S3 Phylogenetic tree of a 399 nucleotide long stretch within the gene coding for p40 (N protein, nucleoprotein). 399 bp corresponding to nt positions 312–710 of reference strain V, GenBank acc. No. U04608. Sequences of BDV-positive bicolored white-toothed shrews are marked with red diamonds [this paper], blue circles [35], [36] and purple triangle [39]. For details of spill-over hosts see reference [61]. For the tree (unrooted tree, 88 nucleotide sequences) the Neighbor-Joining method was used. The percentage of replicates in the bootstrap test (1000 replicates) is shown next to the branches. Values less than 70% are hidden. BW, Baden-Wurttemberg; HE, Hesse; LS, Lower Saxony; SA, Saxony-Anhalt; SH, Schleswig-Holstein; SX, Saxony; TH, Thuringia (Germany); GB, Graubuenden; SG, Sankt Gallen (Switzerland); L, Liechtenstein (The Principality of Liechtenstein).
(TIF)

Table S1 Overview of the shrew collection.
(DOC)

Table S2 Overview of tissue distribution of BDV in selected bicolored white-toothed shrews (*Crocidura leucodon*) determined by RT-PCR.
(DOC)

Table S3 Overview of nucleotide exchanges in BDV sequences obtained from individual white-toothed bicolored shrews. 1824 bp comprising the N, P, X genes of BDV.
(DOC)
