# Supplementary figures and images for: The Bicolored White-Toothed Shrew Crocidura leucodon (HERMANN 1780) Is an Indigenous Host of Mammalian Borna Disease Virus
Source: PLoS One. 2014 Apr 3;9(4):e93659. doi: 10.1371/journal.pone.0093659 (PMC3974811; doi:10.1371/journal.pone.0093659)

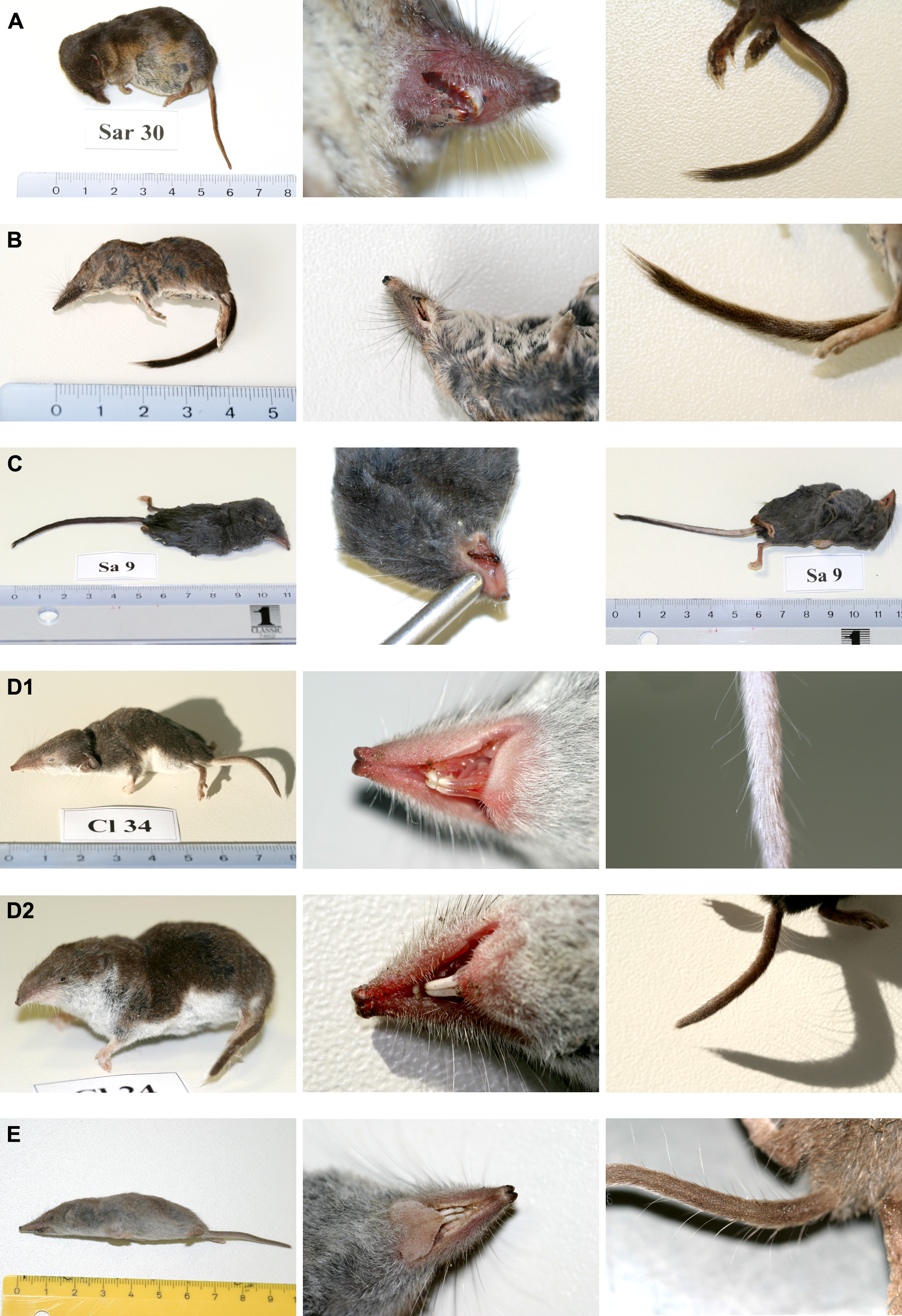

Supplement: Figure S1 — Species identification of shrews investigated in the study. A. Common shrew (Sorex araneus, LINNAEUS 1758); the brown color at the flanks which divides the dark brown back from the grey vent, the red tipped teeth and the smooth tail are typical; B. Pygmy shrew (Sorex minutus, LINNAEUS 1766); the small size and weight (of this individuum 3.405 g) are striking; note the red tipped teeth and the missing lashes at the tail which are characteristics of the genus Sorex; C. Mountain shrew (Sorex alpinus, SCHINZ 1837); the dark color of the body and the long tail are conspicuous; red teeth and the smooth tail are characteristics of the Sorex-species; D1. Bicolored white-toothed shrew (Crocidura leucodon, HERMANN 1780); the bicolored body and the lashes at the tail are striking; the tail is short; members of the genus Crocidura do not possess red tipped teeth; D2: Bicolored white-toothed shrew (Crocidura leucodon, HERMANN 1780); another specimen; note the white color ventral, the white teeth and the lashes under the tail; E. Greater white-toothed shrew (Crocidura russula, HERMANN 1780); this species has a grey vent, the teeth are white, and there are lashes at the tail. (TIF) [file pone.0093659.s001.tif]

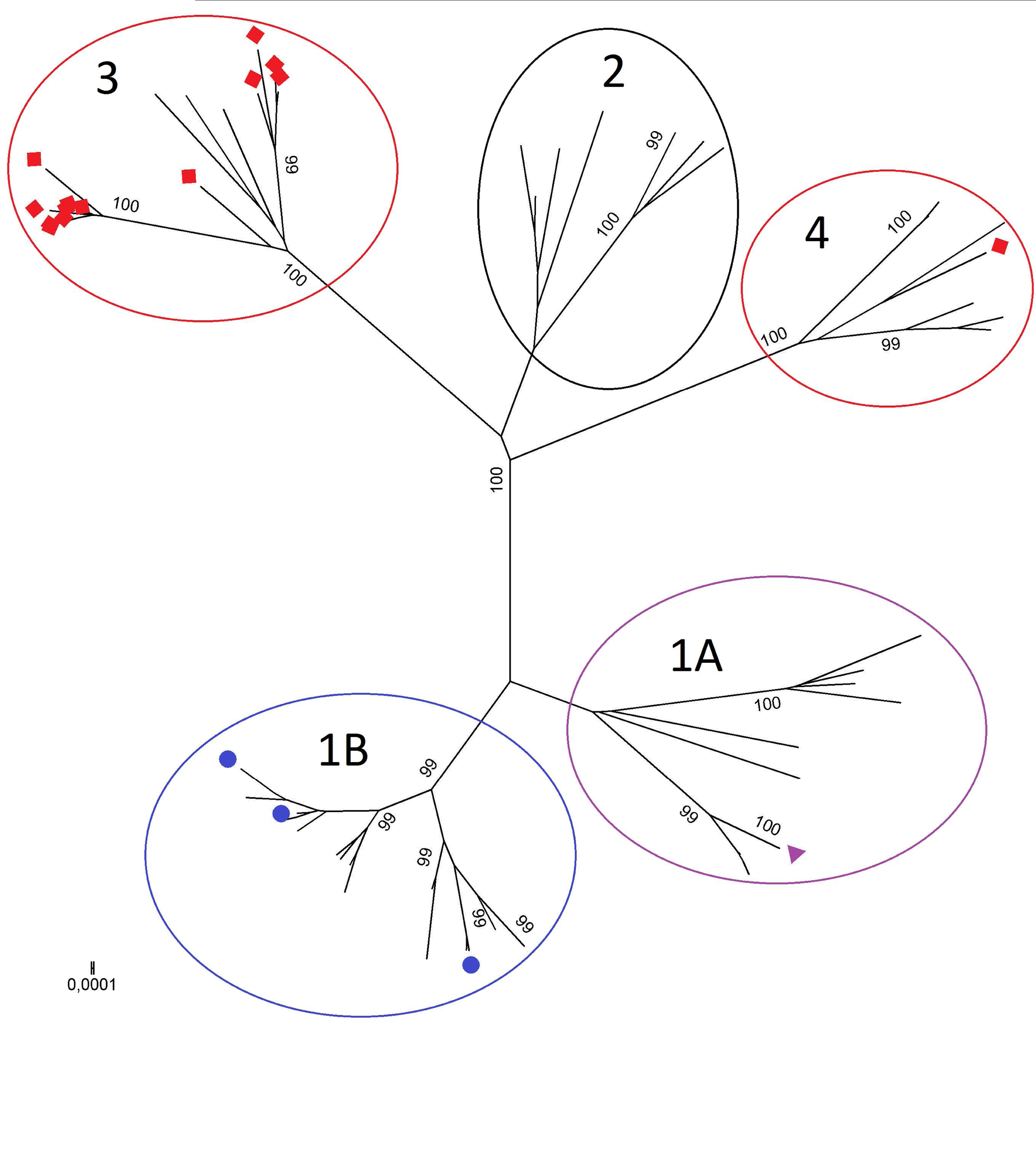

Supplement: Figure S2 — Radial phylogenetic tree representing the position of the BDV sequences obtained from shrews. Sequences of BDV-positive bicolored white-toothed shrews are marked with red diamonds [this paper], blue circles [35], [36] and purple triangle [39]. Sequences of BDVs from spill-over hosts do not have marks. The tree (Neighbor- Joining method) includes a 1824 bp stretch of the BDV genome comprising the N, P, and X genes and consists of 63 sequences. Bootstrap values less than 99% are hidden. (TIF) [file pone.0093659.s002.tif]

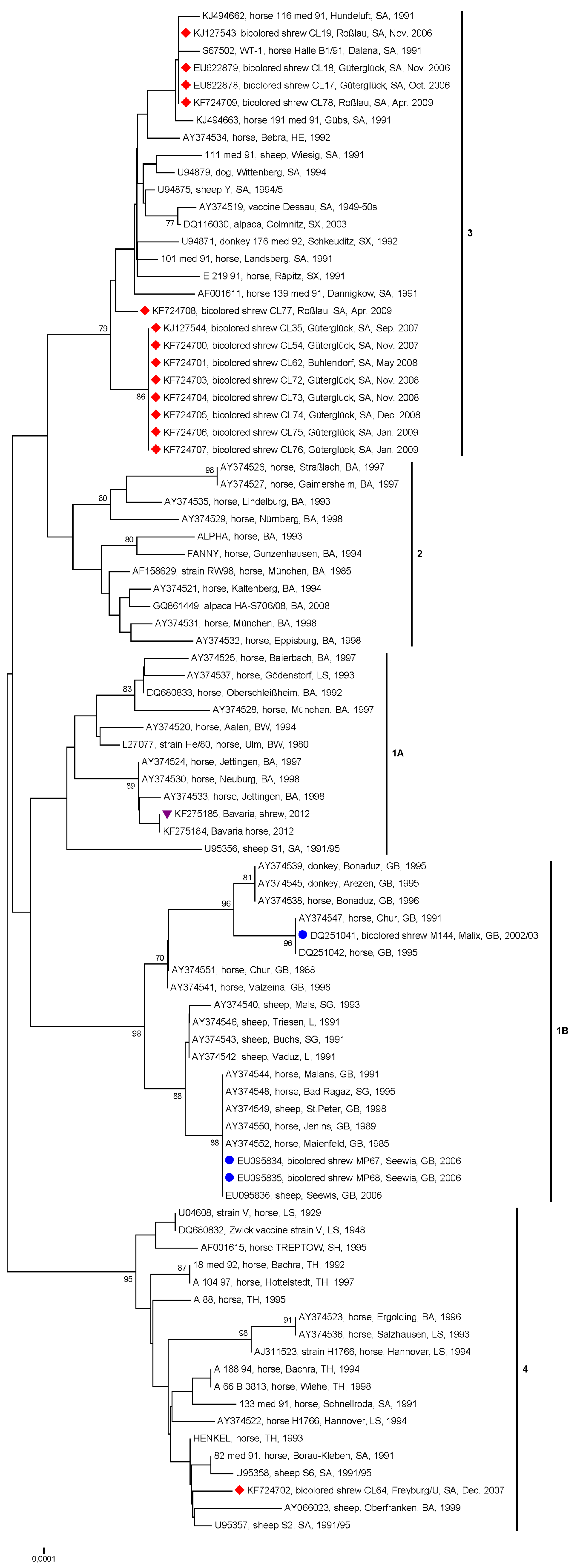

Supplement: Figure S3 — Phylogenetic tree of a 399 nucleotide long stretch within the gene coding for p40 (N protein, nucleoprotein). 399 bp corresponding to nt positions 312–710 of reference strain V, GenBank acc. No. U04608. Sequences of BDV-positive bicolored white-toothed shrews are marked with red diamonds [this paper], blue circles [35], [36] and purple triangle [39]. For details of spill-over hosts see reference [61]. For the tree (unrooted tree, 88 nucleotide sequences) the Neighbor-Joining method was used. The percentage of replicates in the bootstrap test (1000 replicates) is shown next to the branches. Values less than 70% are hidden. BW, Baden-Wurttemberg; HE, Hesse; LS, Lower Saxony; SA, Saxony-Anhalt; SH, Schleswig-Holstein; SX, Saxony; TH, Thuringia (Germany); GB, Graubuenden; SG, Sankt Gallen (Switzerland); L, Liechtenstein (The Principality of Liechtenstein). (TIF) [file pone.0093659.s003.tif]
