# Supplementary material for: The Bicolored White-Toothed Shrew Crocidura leucodon (HERMANN 1780) Is an Indigenous Host of Mammalian Borna Disease Virus
Source: PLoS One. 2014 Apr 3;9(4):e93659. doi: 10.1371/journal.pone.0093659 (PMC3974811; doi:10.1371/journal.pone.0093659)
Supplement: Table S1 — Overview of the shrew collection. (DOC) [file pone.0093659.s004.doc]

**Supplementary Table S1.** Overview of the shrew collection.

| Shrew  no.^a^ | Species | Length in cm | | | Body weight  in g | Location | Date of collection | BDV  positive^b^ |
| --- | --- | --- | --- | --- | --- | --- | --- | --- |
|  |  | total | head +  rump | tail |  |  |  |  |
| Cr 1 | *Crocidura russula* | 9.8 | 6.7 | 3.1 | 6.468 | Hohendodeleben (SA)  52°06'11.30"N, 11°31'32.83"O, 114 m | 20.09.05 | - |
| Cr 2 | *Crocidura russula* | 10.2 | 6.5 | 3.7 | 10.702 | Umpferstedt (TH)  50°58'36.86"N, 11°24'24.69"O, 273 m | before 26.10.05 | - |
| Sar 3 | *Sorex araneus* | 9.2 | 6.0 | 3.2 | 7.715 | Hohendodeleben (SA)  52°06'11.30"N, 11°31'32.83"O, 114 m | before 02.11.05 | - |
| Cl 4 | *Crocidura leucodon* | 9.1 | 6.9 | 2.2 | 7.970 | Güterglück (SA)  51°59'19.28"N, 11°59'44.82"O, 71 m | Nov. 05 | - |
| Cr 5 | *Crocidura russula* | 11.3 | 8.1 | 3.2 | 11.284 | Bad Berka (TH)  50°54'00.73"N, 11°17'06.34"O, 269 m | Dec. 05 | - |
| Cl 6 | *Crocidura leucodon* | 10.8 | 8.0 | 2.8 | 8.426 | Hohendodeleben (SA)  52°06'11.30"N, 11°31'32.83"O, 114 m | Oct. 05 | - |
| Cl 7 | *Crocidura leucodon* | 9.8 | 7.2 | 2.6 | 5.908 | Tornau (SA)  51°54'03.05"N, 12°13'02.62"O, 73 m | 02.02.06 | - |
| Cl 8 | *Crocidura leucodon* | 9.9 | 7.4 | 2.5 | 6.186 | Roßlau (SA)  51°53'08.19"N, 12°14'08.58"O, 70 m | 13.07.06 | - |
| Sa 9 | *Sorex alpinus* | 13.9 | 7.7 | 6.2 | 7.259 | Unterperlhof, Katharinaberg (ST)  46°40'22.39"N, 10°56'55.47"O, 1090 m | 30.07.06 | - |
| Sar 10 | *Sorex araneus* | 11.6 | 7.6 | 4.0 | 7.890 | Roßlau (SA)  51°53'08.19"N, 12°14'08.58"O, 70 m | 14.08.06 | - |
| Sar 11 | *Sorex araneus* | 10.5 | 6.8 | 3.7 | 10.778 | Nossen (S)  51°03'15.48"N, 13°18'37.90"O, 219 m | 28.08.06 | - |
| Cl 12 | *Crocidura leucodon* | 9.5 | 6.5 | 3.0 | 8.383 | Roßlau (SA)  51°53'08.19"N, 12°14'08.58"O, 70 m | 03.10.06 | - |
| Cl 13 | *Crocidura leucodon* | 10.1 | 7.1 | 3.0 | 7.855 | Roßlau (SA)  51°53'08.19"N, 12°14'08.58"O, 70 m | 05.10.06 | - |
| Cr 14 | *Crocidura russula* | 10.5 | 6.8 | 3.7 | 8.639 | Schleberoda (SA)  51°14'N, 11°48'O, 202 m | 13.06.06 | - |
| Cl 15 | *Crocidura leucodon* | 8.9 | 6.4 | 2.5 | 4.126 | Bad Bibra (SA)  51°12'N, 11°35'O, 150 m | 28.09.06 | - |
| Cl 16 | *Crocidura leucodon* | 8.3 | 5.6 | 2.7 | 3.312 | Bad Bibra (SA)  51°12'N, 11°35'O, 150 m | 29.09.06 | - |

TH, Thuringia; S, Saxony; SA, Saxony-Anhalt; ST, South Tyrol; LS, Lower Saxony; ^a^ for better differentiation between letters and numbers upper cases were used in the paper;

^b^ RNA/antigen/genome (integration of nucleoprotein); -, negative; +, positive

**Supplementary Table S1 (continued).** Overview of the shrew collection.

| Shrew  no.^a^ | Species | Length in cm | | | Body weight  in g | Location | Date of collection | BDV  positive^b^ |
| --- | --- | --- | --- | --- | --- | --- | --- | --- |
|  |  | total | head +  rump | tail |  |  |  |  |
| Cl 17 | *Crocidura leucodon* | 10.5 | 7.5 | 3.0 | 8.247 | Güterglück (SA)  51°59'19.28"N, 11°59'44.82"O, 71 m | Oct. 2006 | +/+/+ |
| Cl 18 | *Crocidura leucodon* | 8.7 | 6.0 | 2.7 | 7.953 | Güterglück (SA)  51°59'19.28"N, 11°59'44.82"O, 71 m | Nov. 2006 | +/+/- |
| Cl 19 | *Crocidura leucodon* | 10.3 | 8.1 | 2.2 | 9.287 | Roßlau (SA)  51°53'08.19"N, 12°14'08.58"O, 70 m | Nov. 2006 | +/-/- |
| Cl 20 | *Crocidura leucodon* | 10.5 | 7.9 | 2.6 | 10.790 | Roßlau (SA)  51°53'08.19"N, 12°14'08.58"O, 70 m | Dec. 2006 | - |
| Cl 21 | *Crocidura leucodon* | 8.7 | 6.0 | 2.7 | 9.097 | Güterglück (SA)  51°59'18.35"N, 11°59'44.98"O, 71 m | 19.01.2007 | - |
| Cl 22 | *Crocidura leucodon* | 10.8 | 8.3 | 2.5 | 5.587 | Freyburg/Unstrut (SA)  51°12'31.25"N, 11°46'17.54"O, 107 m | 14.01.2007 | - |
| Cl 23 | *Crocidura leucodon* | 11.1 | 8.3 | 2.9 | 11.9 | Freyburg/Unstrut (SA)  51°12'N, 11°46'O, 107 m | 08.01.2007 | - |
| Cl 24 | *Crocidura leucodon* | 9.8 | 7.4 | 2.4 | 11.665 | Freyburg/Unstrut (SA)  51°12'N, 11°46'O, 107 m | 11.01.2007 | - |
| Cl 25 | *Crocidura leucodon* | 9.9 | 7.4 | 2.5 | 9.687 | Freyburg/Unstrut (SA)  51°12'N, 11°46'O, 107 m | 13.01.2007 |  |
| Cl 26 | *Crocidura leucodon* | 10.5 | 8.0 | 2.5 | 11.027 | Freyburg/Unstrut (SA)  51°12'N, 11°46'O, 107 m | 14.01.2007 | - |
| Cl 27 | *Crocidura leucodon* | 10.8 | 8.5 | 2.3 | 9.045 | Freyburg/Unstrut (SA)  51°12'N, 11°46'O, 107 m | 14.01.2007 | - |
| Cl 28 | *Crocidura leucodon* | 10.3 | 7.4 | 2.9 | 9.472 | Roßlau (SA)  51°53'08.19"N, 12°14'08.58"O, 70 m | 01.03.2007 | - |
| Cl 29 | *Crocidura leucodon* | 10.2 | 7.5 | 2.7 | 7.755 | Roßlau (SA)  51°53'08.19"N, 12°14'08.58"O, 70 m | 25.05.2007 | - |
| Sar 30 | *Sorex araneus* | 12.5 | 8.8 | 3.7 | 11.775 | Roßlau (SA)  51°53'08.19"N, 12°14'08.58"O, 70 m | 14.07.2007 | - |
| Sar 31 | *Sorex araneus* | 11.7 | 7.5 | 4.2 | 6.615 | Roßlau (SA)  51°53'08.19"N, 12°14'08.58"O, 70 m | Aug. 2007 | - |
| Cl 32 | *Crocidura leucodon* | 11.0 | 8.8 | 2.2 | 6.362 | Roßlau (SA)  51°53'08.19"N, 12°14'08.58"O, 70 m | Aug. 2007 | - |

TH, Thuringia; S, Saxony; SA, Saxony-Anhalt; ST, South Tyrol; LS, Lower Saxony; ^a^ for better differentiation between letters and numbers upper cases were used in the paper;

^b^ RNA/antigen/genome (integration of nucleoprotein); -, negative; +, positive

**Supplementary Table S1 (continued).** Overview of the shrew collection.

| Shrew  no.^a^ | Species | Length in cm | | | Body weight  in g | Location | Date of collection | BDV  positive^b^ |
| --- | --- | --- | --- | --- | --- | --- | --- | --- |
|  |  | total | head +  rump | tail |  |  |  |  |
| Sar 33 | *Sorex araneus* | 11.0 | 7.2 | 3.8 | 10.551 | Roßlau (SA)  51°53'08.19"N, 12°14'08.58"O, 70 m | 19.09.2007 | - |
| Cl 34 | *Crocidura leucodon* | 10.0 | 7.2 | 2.8 | 7.841 | Güterglück (SA)  51°59'19.28"N, 11°59'44.82"O, 71 m | 19.09.2007 | - |
| Cl 35 | *Crocidura leucodon* | 9.5 | 6.7 | 2.8 | 7.150 | Güterglück (SA)  51°59'19.28"N, 11°59'44.82"O, 71 m | 19.09.2007 | +/-/- |
| Sar 36 | *Sorex araneus* | 10.9 | 7.4 | 3.5 | 11.979 | Roßlau (SA)  51°53'08.19"N, 12°14'08.58"O, 70 m | 29.09.2007 | - |
| Cl 37 | *Crocidura leucodon* | 9.4 | 6.5 | 2.9 | 6.555 | Roßlau (SA)  51°53'08.19"N, 12°14'08.58"O, 70 m | 14.10.2007 | - |
| Cl 38 | *Crocidura leucodon* | 10.6 | 8.2 | 2.4 | 8.515 | Güterglück (SA)  51°59'19.28"N, 11°59'44.82"O, 71 m | Oct. 2007 | - |
| Cl 39 | *Crocidura leucodon* | 10.7 | 8.3 | 2.4 | 8.691 | Güterglück (SA)  51°59'19.28"N, 11°59'44.82"O, 71 m | Oct. 2007 | - |
| Cl 40 | *Crocidura leucodon* | 10.8 | 7.8 | 3.0 | 9.113 | Schleberoda (SA)  51°14'N, 11°48'O, 202 m | 16.06.2007 | - |
| Cl 41 | *Crocidura leucodon* | 10.6 | 8.2 | 2.4 | 8.158 | Schleberoda (SA)  51°14'N, 11°48'O, 202 m | 16.06.2007 | - |
| Cl 42 | *Crocidura leucodon* | 11.1 | 8.4 | 2.7 | 10.327 | Schleberoda (SA)  51°14'N, 11°48'O, 202 m | 17.06.2007 | - |
| Cr 43 | *Crocidura russula* | 12.7 | 9.3 | 3.4 | 13.229 | Schleberoda (SA)  51°14'N, 11°48'O, 202 m | 20.06.2007 | - |
| Sm 44 | *Sorex minutus* | 8.6 | 5.6 | 3.0 | 3.405 | Mücheln-Biendorf, Feldmark (SA)  51°18'N, 11°49'"O, 161 m | 09.07.2007 | - |
| Cl 45 | *Crocidura leucodon* | 11.1 | 8.3 | 2.8 | 8.118 | Schleberoda (SA)  51°14'N, 11°48'O, 202 m | 12.07.2007 | - |
| Cr 46 | *Crocidura russula* | 10.9 | 7.5 | 3.4 | 5.901 | Freyburg, Eckstädter Gärten (SA)  51°12'41.63"N, 11°46'02.19"O, 104 m | 25.07.2007 | - |
| Sar 47 | *Sorex araneus* | 10.4 | 6.6 | 3.8 | 7.010 | Schleberoda (SA)  51°14'N, 11°48'O, 202 m | 31.07.2007 | - |
| Cr 48 | *Crocidura russula* | 12.6 | 8.5 | 4.1 | 10.319 | Mücheln (SA)  51°15'21.97"N, 11°51'20.82"O, 131 m | 18.07.2007 | - |

TH, Thuringia; S, Saxony; SA, Saxony-Anhalt; ST, South Tyrol; LS, Lower Saxony; ^a^ for better differentiation between letters and numbers upper cases were used in the paper;

^b^ RNA/antigen/genome (integration of nucleoprotein); -, negative; +, positive

**Supplementary Table S1 (continued).** Overview of the shrew collection.

| Shrew  no.^a^ | Species | Length in cm | | | Body weight  in g | Location | Date of collection | BDV  positive^b^ |
| --- | --- | --- | --- | --- | --- | --- | --- | --- |
|  |  | total | head +  rump | tail |  |  |  |  |
| Cr 49 | *Crocidura russula* | 10.8 | 7.3 | 3.5 | 9.167 | Schleberoda (SA)  51°14'N, 11°48'O, 202 m | 17.08.2007 | - |
| Cl 50 | *Crocidura leucodon* | 11.9 | 8.3 | 3.6 | 10.835 | Schleberoda (SA)  51°14'N, 11°48'O, 202 m | 17.10.2007 | - |
| Cl 51 | *Crocidura leucodon* | 10.5 | 7.3 | 3.2 | 9.301 | Schleberoda (SA)  51°14'N, 11°48'O, 202 m | 20.10.2007 | - |
| Cl 52 | *Crocidura leucodon* | 12.0 | 8.5 | 3.5 | 10.558 | Schleberoda (SA)  51°14'N, 11°48'O, 202 m | 20.10.2007 | - |
| Cl 53 | *Crocidura leucodon* | 8.8 | 6.5 | 2.3 | 7.791 | Güterglück (SA)  51°59'19.28"N, 11°59'44.82"O, 71 m | Nov. 2007 | - |
| Cl 54 | *Crocidura leucodon* | 8.7 | 6.4 | 2.3 | 7.044 | Güterglück (SA)  51°59'19.28"N, 11°59'44.82"O, 71 m | Nov. 2007 | +/+/+ |
| Cl 55 | *Crocidura leucodon* | 9.0 | 6.4 | 2.6 | 8.530 | Güterglück (SA)  51°59'19.28"N, 11°59'44.82"O, 71 m | Nov. 2007 | - |
| Cl 56 | *Crocidura leucodon* | 9.8 | 7.5 | 2.3 | 7.186 | Güterglück (SA)  51°59'19.28"N, 11°59'44.82"O, 71 m | 29.12.2007 | - |
| Cl 57 | *Crocidura leucodon* | 9.4 | 7.0 | 2.4 | 7.543 | Tornau (SA)  51°54'02.19"N, 12°13'04.49"O, 75 m | 04.01.2008 | - |
| Cl 58 | *Crocidura leucodon* | 9.6 | 7.0 | 2.6 | 8.737 | Güterglück (SA)  51°59'17.13"N, 11°59'48.09"O, 71 m | 12.01.2008 | - |
| Cl 59 | *Crocidura leucodon* | 8.9 | 6.7 | 2.2 | 6.220 | Moritzer Mühle (SA)  52°00'14.20"N, 12°01'42.92"O, 77 m | 15.01.2008 | - |
| Cl 60 | *Crocidura leucodon* | 9.2 | 6.6 | 2.6 | 7.845 | Güterglück (SA)  51°59'02.60"N, 11°59'38.77"O, 70 m | 18.01.2008 | - |
| Cl 61 | *Crocidura leucodon* | 8.8 | 6.8 | 2.0 | 10.196 | Meinsdorf (SA)  51°54'26.12"N, 12°15'19.30"O, 66 m | 16.04.2008 | - |
| Cl 62 | *Crocidura leucodon* | 9.9 | 7.2 | 2.7 | 10.751 | Buhlendorf (SA)  52°01'53.42"N, 12°01'52.89"O, 87 m | 07.05.2008 | +/+/- |
| Cl 63 | *Crocidura leucodon* | 10.5 | 7.2 | 3.3 | 7.844 | Freyburg/Unstrut (SA)  51°13'N, 11°46'O, 139 m | 25.12.2007 | - |
| Cl 64 | *Crocidura leucodon* | 10.4 | 7.3 | 3.1 | 7.739 | Freyburg/Unstrut (SA)  51°13'N, 11°46'O, 139 m | 29.12.2007 | +/+/+ |

TH, Thuringia; S, Saxony; SA, Saxony-Anhalt; ST, South Tyrol; LS, Lower Saxony; ^a^ for better differentiation between letters and numbers upper cases were used in the paper;

^b^ RNA/antigen/genome (integration of nucleoprotein); -, negative; +, positive

**Supplementary Table S1 (continued).** Overview of the shrew collection.

| Shrew  no.^a^ | Species | Length in cm | | | Body weight  in g | Location | Date of collection | BDV  positive^b^ |
| --- | --- | --- | --- | --- | --- | --- | --- | --- |
|  |  | total | head +  rump | tail |  |  |  |  |
| Cr 65 | *Crocidura russula* | 11.4 | 7.5 | 3.9 | 8.585 | Braunsbedra (SA)  51°17'N, 11°53'O, 130 m | 20.04.2008 | - |
| Sar 66 | *Sorex araneus* | 11.3 | 7.5 | 3.8 | 6.571 | Sorge/Harz (SA)  51°41'N, 10°42'O, 511 m | 29.06.2008 | - |
| Sar 67 | *Sorex araneus* | 12.9 | 9.3 | 3.6 | 6.869 | Sorge/Harz (SA)  51°41'N, 10°42'O, 511 m | 29.06.2008 | - |
| Sar 68 | *Sorex araneus* | 11.4 | 7.7 | 3.7 | 9.917 | Roßlau (SA)  51°53'08.19"N, 12°14'08.58"O, 70 m | 29.06.2008 | - |
| Sar 69 | *Sorex araneus* | 11.5 | 7.2 | 4.3 | 5.724 | Bad Berka (TH)  50°54'00.73"N, 11°17'06.34"O, 269 m | Aug. 2007 | - |
| Cr 70 | *Crocidura russula* | 11.2 | 7.8 | 3.4 | 9.612 | Wittingen (LS)  52°43'36.19"N, 10°43'56.60"O, 84 m | 11.09.2008 | - |
| Sar 71 | *Sorex araneus* | 10.0 | 6.5 | 3.5 | 7.405 | Roßlau (SA)  51°53'08.19"N, 12°14'08.58"O, 70 m | 08.11.2008 | - |
| Cl 72 | *Crocidura leucodon* | 9.0 | 7.0 | 2.0 | 6.796 | Güterglück (SA)  51°59'19.28"N, 11°59'44.82"O, 71 m | 18.11.2008 | +/+/- |
| Cl 73 | *Crocidura leucodon* | 9.5 | 6.9 | 2.6 | 7.624 | Güterglück (SA)  51°59'19.28"N, 11°59'44.82"O, 71 m | 21.11.2008 | +/+/- |
| Cl 74 | *Crocidura leucodon* | 9.0 | 6.5 | 2.5 | 8.314 | Güterglück (SA)  51°59'19.28"N, 11°59'44.82"O, 71 m | 22.12.2008 | +/+/- |
| Cl 75 | *Crocidura leucodon* | 9.1 | 6.5 | 2.6 | 8.349 | Güterglück (SA)  51°59'19.28"N, 11°59'44.82"O, 71 m | 23.01.2009 | +/+/- |
| Cl 76 | *Crocidura leucodon* | 9.7 | 7.0 | 2.7 | 9.337 | Güterglück (SA)  51°59'19.28"N, 11°59'44.82"O, 71 m | 24.01.2009 | +/+/- |
| Cl 77 | *Crocidura leucodon* | 7.7 | 4.9 | 2.8 | 10.098 | Roßlau (SA)  51°53'08.19"N, 12°14'08.58"O, 70 m | 19.04.2009 | +/+/- |
| Cl 78 | *Crocidura leucodon* | 7.5 | 4.9 | 2.6 | 9.397 | Roßlau (SA)  51°53'08.19"N, 12°14'08.58"O, 70 m | 28.04.2009 | +/+/- |
| Cr 79 | *Crocidura russula* | 10.9 | 7.0 | 3.9 | 6.902 | Schleberoda (SA)  51°14'N, 11°48'O, 202 m | 03.02.2008 | - |
| Cr 80 | *Crocidura russula* | 12.0 | 8.5 | 3.5 | 10.699 | Schleberoda (SA)  51°14'N, 11°48'O, 202 m | 03.02.2008 | - |

TH, Thuringia; S, Saxony; SA, Saxony-Anhalt; ST, South Tyrol; LS, Lower Saxony; ^a^ for better differentiation between letters and numbers upper cases were used in the paper;

^b^ RNA/antigen/genome (integration of nucleoprotein); -, negative; +, positive

**Supplementary Table S1 (continued).** Overview of the shrew collection.

| Shrew  no.^a^ | Species | Length in cm | | | Body weight  in g | Location | Date of collection | BDV  positive^b^ |
| --- | --- | --- | --- | --- | --- | --- | --- | --- |
|  |  | total | head +  rump | tail |  |  |  |  |
| Cr 81 | *Crocidura russula* | 11.3 | 7.7 | 3.6 | 9.521 | Schleberoda (SA)  51°14'N, 11°48'O, 202 m | 03.02.2008 | - |
| Cr 82 | *Crocidura russula* | 11.9 | 8.6 | 3.3 | 10.279 | Schleberoda (SA)  51°14'N, 11°48'O, 202 m | 03.02.2008 | - |
| Cr 83 | *Crocidura russula* | 11.8 | 8.4 | 3.4 | 9.386 | Schleberoda (SA)  51°14'N, 11°48'O, 202 m | 03.02.2008 | - |
| Cr 84 | *Crocidura russula* | 12.1 | 8.6 | 3.5 | 9.112 | Freyburg/Unstrut, Ehrenberge (SA)  51°13'N, 11°46'O, 139 m | 18.09.2008 | - |
| Cr 85 | *Crocidura russula* | 11.6 | 8.4 | 3.2 | 11.180 | Schleberoda (SA)  51°14'19.97"N, 11°48'08.39"O, 202 m | 2008 | - |
| Sm 86 | *Sorex minutus* | 8.7 | 5.0 | 3.7 | 1.745 | Freyburg/Unstrut (SA)  51°13'N, 11°46'O, 139 m | 2008 | - |
| Cr 87 | *Crocidura russula* | 10.5 | 7.0 | 3.5 | 6.9 | Freyburg/Unstrut (SA)  51°13'N, 11°46'O, 139 m | 2008 | - |
| Cl 88 | *Crocidura leucodon* | 7.0 | 4.5 | 2.5 | 3.263 | Freyburg/Unstrut (SA)  51°13'N, 11°46'O, 139 m | 2008 | - |
| Cr 89 | *Crocidura russula* | 11.8 | 7.8 | 4.0 | 10.358 | Freyburg/Unstrut (SA)  51°13'N, 11°46'O, 139 m | 2008 | - |
| Cr 90 | *Crocidura russula* | 10.4 | 7.2 | 3.2 | 8.897 | Freyburg/Unstrut (SA)  51°13'N, 11°46'O, 139 m | 2008 | - |
| Sar 91 | *Sorex araneus* | 10.6 | 7.1 | 3.5 | 10.360 | Bad Berka, Arbelsberg (TH)  50°53'N, 11°16'O, 351 m | 29.05.2009 | - |
| Cl 92 | *Crocidura leucodon* | 11.3 | 8.6 | 2.7 | 7.225 | Güterglück (SA)  51°59'19.28"N, 11°59'44.82"O, 71 m | 16.08.2009 | - |
| Cl 93 | *Crocidura leucodon* | 11.4 | 8.3 | 3.1 | 5.975 | Meinsdorf (SA)  51°54'11.52"N, 12°15'11.10"O, 71 m | 18.08.2009 | - |
| Sar 94 | *Sorex araneus* | 12.0 | 8.5 | 3.5 | 9.429 | Leitzkau (SA)  52°03'N, 11°57'O, 107 m | 15.09.2009 | - |
| Cl 95 | *Crocidura leucodon* | 9.6 | 7.3 | 2.3 | 8.461 | Meinsdorf (SA)  51°54'26.12"N, 12°15'19.30"O, 66 m | 01.11.2009 | - |
| Cr 96 | *Crocidura russula* | 11.1 | 8.0 | 3.1 | 8.774 | Wittingen (LS)  52°43'36.19"N, 10°43'56.60"O, 84 m | 21.01.2009 | - |

TH, Thuringia; S, Saxony; SA, Saxony-Anhalt; ST, South Tyrol; LS, Lower Saxony; ^a^ for better differentiation between letters and numbers upper cases were used in the paper;

^b^ RNA/antigen/genome (integration of nucleoprotein); -, negative; +, positive

**Supplementary Table S1 (continued).** Overview of the shrew collection.

| Shrew  no.^a^ | Species | Length in cm | | | Body weight  in g | Location | Date of collection | BDV  positive^b^ |
| --- | --- | --- | --- | --- | --- | --- | --- | --- |
|  |  | total | head +  rump | tail |  |  |  |  |
| Cr 97 | *Crocidura russula* | 12.7 | 9.0 | 3.7 | 7.909 | Wittingen (LS)  52°43'36.19"N, 10°43'56.60"O, 84 m | 15.04.2009 | - |
| Cr 98 | *Crocidura russula* | 12.4 | 8.4 | 4.0 | 10.880 | Wittingen (LS)  52°43'36.19"N, 10°43'56.60"O, 84 m | 15.06.2009 | - |
| Cr 99 | *Crocidura russula* | 11.9 | 8.5 | 3.4 | 7.908 | Wittingen (LS)  52°43'36.19"N, 10°43'56.60"O, 84 m | 05.08.2009 | - |
| Cr 100 | *Crocidura russula* | 11.2 | 7.5 | 3.7 | 10.750 | Wittingen (LS)  52°43'36.19"N, 10°43'56.60"O, 84 m | 05.08.2009 | - |
| Cr 101 | *Crocidura russula* | 11.9 | 8.5 | 3.4 | 7.493 | Roßlau (SA)  51°53'08.19"N, 12°14'08.58"O, 70 m | 22.08.2010 | - |
| Cl 102 | *Crocidura leucodon* | 9.6 | 7.0 | 2.6 | 6.324 | Meinsdorf Garten (SA)  51°54'26.12"N, 12°15'19.30"O, 66 m | 12.10.2010 | - |
| Sar103 | *Sorex araneus* | 11.8 | 7.6 | 4.2 | 10.0 | Weischütz (SA)  51°13'15.13"N, 11°42'28.30"O, 116 m | 04.04.2009 | - |
| Cr 104 | *Crocidura russula* | 11.6 | 7.7 | 3.9 | 8.0 | Weischütz (SA)  51°13'15.13"N, 11°42'28.30"O, 116 m | 22.09.2009 | - |
| Sm105 | *Sorex minutus* | 9.9 | 6.5 | 3.4 | 4.0 | Weischütz (SA)  51°13'15.13"N, 11°42'28.30"O, 116 m | 16.08.2009 | - |
| Sm106 | *Sorex minutus* | 9.2 | 5.4 | 3.8 | 4.0 | Weischütz (SA)  51°13'15.13"N, 11°42'28.30"O, 116 m | 17.09.2009 | - |
| Cr 107 | *Crocidura russula* | 10.6 | 7.2 | 3.4 | 12.0 | Schleberoda (SA)  51°14'20.08"N, 11°48'06.01"O, 203 m | 05.12.2010 | - |

TH, Thuringia; S, Saxony; SA, Saxony-Anhalt; ST, South Tyrol; LS, Lower Saxony; ^a^ for better differentiation between letters and numbers upper cases were used in the paper;

^b^ RNA/antigen/genome (integration of nucleoprotein); -, negative; +, positive
