# Supplementary material for: The Bicolored White-Toothed Shrew Crocidura leucodon (HERMANN 1780) Is an Indigenous Host of Mammalian Borna Disease Virus
Source: PLoS One. 2014 Apr 3;9(4):e93659. doi: 10.1371/journal.pone.0093659 (PMC3974811; doi:10.1371/journal.pone.0093659)
Supplement: Table S2 — Overview of tissue distribution of BDV in selected bicolored white-toothed shrews (Crocidura leucodon) determined by RT-PCR. (DOC) [file pone.0093659.s005.doc]

**Supplementary Table S2.** Overview of tissue distribution of BDV in selected bicolored white-toothed shrews (*Crocidura leucodon*) determined by RT-PCR.

| Shrew no. | Tissue | BDV RT-PCR p40 | BDV RT-PCR p24 |
| --- | --- | --- | --- |
| Cl 17 | Brain | **+** | **+** |
|  | Liver | **+** | **+** |
|  | Lung | **+** | **+** |
|  | Kidney | **+** | **+** |
|  | Intestine | **+** | **+** |
|  | Bladder | **+** | **+** |
| Cl 18 | Brain | **+** | **+** |
|  | Liver | **+** | **+** |
|  | Lung | **+** | **+** |
|  | Spleen | **+** | **+** |
|  | Heart | **+** | **+** |
|  | Stomach | **+** | **+** |
|  | Intestine | **+** | **+** |
|  | Bladder | **+** | **+** |
| Cl 19 | Brain | **+** | **+** |
|  | Liver | - | - |
|  | Lung | - | - |
|  | Kidney | - | - |
|  | Stomach | - | **+** |
|  | Intestine | - | - |
|  | Bladder | - | - |
|  | Testis | - | - |
|  | Heart | - | - |
| Cl 35 | Brain | **+** | **+** |
|  | Liver | - | - |
|  | Lung | **(+)** | **(+)** |
|  | Kidney | - | - |
|  | Intestine | - | - |
|  | Testis | - | - |
|  | Nose mucosa | - | - |

**+** positive; **(+)** weak reaction; - negative
