# Supplementary material for: The Bicolored White-Toothed Shrew Crocidura leucodon (HERMANN 1780) Is an Indigenous Host of Mammalian Borna Disease Virus
Source: PLoS One. 2014 Apr 3;9(4):e93659. doi: 10.1371/journal.pone.0093659 (PMC3974811; doi:10.1371/journal.pone.0093659)
Supplement: Table S3 — Overview of nucleotide exchanges in BDV sequences obtained from individual white-toothed bicolored shrews. 1824 bp comprising the N, P, X genes of BDV. (DOC) [file pone.0093659.s006.doc]

**Supplementary Table S3.** Overview of nucleotide exchanges in BDV sequences obtained from individual white-toothed bicolored shrews (1824 bp comprising the N, P, X genes of BDV).

|  | **CL17** | **CL18** | **CL19** | **CL35** | **CL54** | **CL62** | **CL64** | **CL72** | **CL73** | **CL74** | **CL75** | **CL76** | **CL77** | **CL78** | Acc. no.  GenBank |
| --- | --- | --- | --- | --- | --- | --- | --- | --- | --- | --- | --- | --- | --- | --- | --- |
| **CL17** | - | 10 | 7 | 26 | 26 | 28 | 64 | 26 | 27 | 25 | 27 | 31 | 18 | 11 | EU622878 |
| **CL18** | 10 | - | 3 | 26 | 26 | 28 | 66 | 26 | 27 | 25 | 27 | 31 | 20 | 13 | EU622879 |
| **CL19** | 7 | 3 | - | 25 | 25 | 27 | 65 | 25 | 26 | 24 | 26 | 30 | 19 | 12 | KJ127543 |
| **CL35** | 26 | 26 | 25 | - | 2 | 4 | 68 | 2 | 1 | 1 | 1 | 7 | 20 | 29 | KJ127544 |
| **CL54** | 26 | 26 | 25 | 2 | - | 4 | 68 | 2 | 3 | 1 | 3 | 7 | 20 | 29 | KF724700 |
| **CL62** | 28 | 28 | 27 | 4 | 4 | - | 70 | 4 | 5 | 3 | 5 | 9 | 22 | 31 | KF724701 |
| **CL64** | 64 | 66 | 65 | 68 | 68 | 70 | - | 68 | 69 | 67 | 69 | 72 | 60 | 67 | KF724702 |
| **CL72** | 26 | 26 | 25 | 2 | 2 | 4 | 68 | - | 3 | 1 | 3 | 7 | 20 | 29 | KF724703 |
| **CL73** | 27 | 27 | 26 | 1 | 3 | 5 | 69 | 3 | - | 2 | 0 | 8 | 21 | 30 | KF724704 |
| **CL74** | 25 | 25 | 24 | 1 | 1 | 3 | 67 | 1 | 2 | - | 2 | 6 | 19 | 28 | KF724705 |
| **CL75** | 27 | 27 | 26 | 1 | 3 | 5 | 69 | 3 | 0 | 2 | - | 8 | 21 | 30 | KF724706 |
| **CL76** | 31 | 31 | 30 | 7 | 7 | 9 | 72 | 7 | 8 | 6 | 8 | - | 25 | 32 | KF724707 |
| **CL77** | 18 | 20 | 19 | 20 | 20 | 22 | 60 | 20 | 21 | 19 | 21 | 25 | - | 21 | KF724708 |
| **CL78** | 11 | 13 | 12 | 29 | 29 | 31 | 67 | 29 | 30 | 28 | 30 | 32 | 21 | - | KF724709 |
| Location | Güter-glück | Güter-glück | Roßlau | Güter-glück | Güter-glück | Buhlen-dorf | Frey-burg/U. | Güter-glück | Güter-glück | Güter-glück | Güter-glück | Güter-glück | Roßlau | Roßlau |  |
| Collection time | Oct  2006 | Nov  2006 | Nov  2006 | Sep  2007 | Nov  2007 | May  2008 | Dec  2007 | Nov  2008 | Nov  2008 | Dec  2008 | Jan  2009 | Jan  2009 | Apr  2009 | Apr  2009 |  |
